# Chitin scaffolds derived from the marine demosponge *Aplysina fistularis* stimulate the differentiation of dental pulp stem cells

**DOI:** 10.3389/fbioe.2023.1254506

**Published:** 2023-11-14

**Authors:** Anna Zawadzka-Knefel, Agnieszka Rusak, Monika Mrozowska, Tomasz Machałowski, Andrzej Żak, Katarzyna Haczkiewicz-Leśniak, Michał Kulus, Piotr Kuropka, Marzenna Podhorska-Okołów, Katarzyna Skośkiewicz-Malinowska

**Affiliations:** ^1^ Department of Conservative Dentistry with Endodontics, Wroclaw Medical University, Wroclaw, Poland; ^2^ Division of Histology and Embryology, Department of Human Morphology and Embryology, Faculty of Medicine, Wroclaw Medical University, Wroclaw, Poland; ^3^ Institute of Chemical Technology and Engineering, Faculty of Chemical Technology, Poznan University of Technology, Poznan, Poland; ^4^ Electron Microscopy Laboratory, Faculty of Chemistry, Wroclaw University of Science and Technology, Wroclaw, Poland; ^5^ Division of Ultrastructural Research, Faculty of Medicine, Wroclaw Medical University, Wroclaw, Poland; ^6^ Division of Histology and Embryology, Department of Biostructure and Animal Physiology, Faculty of Veterinary Medicine, Wroclaw University of Environmental and Life Sciences, Wroclaw, Poland

**Keywords:** dental pulp stem cells, osteoblasts, odontoblasts, chitin, scaffolds

## Abstract

The use of stem cells for tissue regeneration is a prominent trend in regenerative medicine and tissue engineering. In particular, dental pulp stem cells (DPSCs) have garnered considerable attention. When exposed to specific conditions, DPSCs have the ability to differentiate into osteoblasts and odontoblasts. Scaffolds are critical for cell differentiation because they replicate the 3D microenvironment of the niche and enhance cell adhesion, migration, and differentiation. The purpose of this study is to present the biological responses of human DPSCs to a purified 3D chitin scaffold derived from the marine demosponge *Aplysina fistularis* and modified with hydroxyapatite (HAp). Responses examined included proliferation, adhesion, and differentiation. The control culture consisted of the human osteoblast cell line, hFOB 1.19. Electron microscopy was used to examine the ultrastructure of the cells (transmission electron microscopy) and the surface of the scaffold (scanning electron microscopy). Cell adhesion to the scaffolds was determined by neutral red and crystal violet staining methods. An alkaline phosphatase (ALP) assay was used for assessing osteoblast/odontoblast differentiation. We evaluated the expression of osteogenic marker genes by performing ddPCR for ALP, RUNX2, and SPP1 mRNA expression levels. The results show that the chitin biomaterial provides a favorable environment for DPSC and hFOB 1.19 cell adhesion and supports both cell proliferation and differentiation. The chitin scaffold, especially with HAp modification, isolated from *A. fistularis* can make a significant contribution to tissue engineering and regenerative medicine.

## 1 Introduction

The potential of stem cells in tissue regeneration has been one of the most exciting trends in regenerative medicine and tissue engineering in recent years. Many studies have demonstrated the ability of dental pulp stem cells (DPSCs) to differentiate into cell lineages such as endothelial cells, neurons, chondrocytes, adipocytes, osteoblasts, and odontoblasts (Hilkens et al., 2017; Salvador-Clavell et al., 2021). Particular focus is placed on using stem cells for bone regeneration due to the frequent limitations of therapeutic strategies used in injuries, infections, or irreversible bone damage where spontaneous repair is not possible (Morad et al., 2013).

Stem cells are a population of undifferentiated embryonic or adult postnatal cells that have extensive proliferative potential. They are capable of self-renewal and have the ability to differentiate into multiple cell types (Biehl and Russel, 2009). Currently, bone marrow is considered to be the most common and longest utilized adult source of mesenchymal stem cells (BMSCs) (Pittenger et al., 2019). However, using BMSCs has some limitations related to the low survival rate, limited differentiation potential, and complications during bone marrow aspiration (Mohamed-Ahmed et al., 2018). Many studies suggest that oral tissue-derived cells are an important source for autologous cell therapies and may be an alternative to BMSCs (Sakai et al., 2012; Song et al., 2017; Fujii et al., 2018). Till date, various MSC-like cells have been isolated from the oral cavity such as dental pulp stem cells (DPSCs), stem cells from human exfoliated deciduous teeth (SHED), stem cells from apical papilla (SCAP), dental follicle stem cells (DFSCs), periodontal ligament stem cells (PDLSCs), gingiva-derived mesenchymal stem cells (GMSCs), and buccal fat pad-derived stem cells (BFPSCs) (Roato et al., 2021; Qiu et al., 2020).

DPSCs represent an easily accessible source of stem cells in the human mouth (Ledesma-Martínez et al., 2016), and they are located in the dental pulp and are a source of odontoblasts—cells that produce dentin (Rusu et al., 2014). DPSCs are characterized by self-renewal capacity, colony formation (Al and Ashraf, 2021), high proliferation rates (Li et al., 2014), immunomodulatory properties, and multilineage differentiation potential. The most common sources for the collection of human DPSCs are the third molar, the premolar (Staniowski et al. (2021), and the supernumerary permanent teeth removed due to impaction (Salehi et al., 2018), irreversible pulp inflammation, or periodontitis (Staniowski et al. (2021).

Research shows that DPSCs can differentiate into functional osteoblasts *in vitro* and can produce an extracellular and mineralized matrix, as well as live autologous fibrous bone (LAB) tissue (Mortada and Mortada, 2018). Due to this ability, DPSCs can be used in bone regenerative medicine for the treatment of bone defects resulting from neoplastic diseases, congenital deformities, injuries, osteoporosis, iatrogenic effects (surgery), and periodontal diseases (Rusu et al., 2014; Sato et al., 2020).

The differentiation of DPSCs into osteoblasts requires appropriate culture conditions. Exemplary culture media include dexamethasone, ascorbic acid, and glyceryl 10 beta-phosphate. Environmental factors can also influence the initiation of stem cell differentiation (Mielan et al., 2021). In addition to biological factors and growth factors, scaffolds play an important role in tissue engineering and regenerative medicine. They allow three-dimensional cell growth, and the resulting cells form tissues that can be implanted in the body to treat a disease or repair a defect. In biomaterial development, scaffolds must meet several requirements, including biodegradability, non-antigenicity, non-carcinogenicity, non-toxicity, non-mutagenicity, and high biocompatibility with cells (Chan and Leong, 2008). Scaffolds should not only mimic the 3D niche microenvironment but also be able to enhance cell functions such as adherence, differentiation, migration, autocrine production of bioactive factors, growth factors, and immunomodulators (Zhao et al., 2021).

Chitin, as a natural biopolymer commonly found in various organisms around the world, appears to be an excellent biomaterial for use in tissue engineering. As a result of its remarkable mechanical properties (Machałowski et al., 2022; Duminis et al., 2023), non-toxic nature, and ability to form renewable, hierarchical, three-dimensional spatial structures, it creates an optimal environment to promote stem cell viability, proliferation, and differentiation (Wan and Tai, 2013), and research shows that natural biopolymers are similar to the natural extracellular matrix. After implantation, they can reduce the immune response and chronic inflammation (Pina et al., 2019), and chitin exhibits good hemocompatibility and has a cohesive interaction with blood components, such as fibrinogen, albumin, IgG, and erythrocytes, which can enhance therapeutic efficacy (Barzic and Albu, 2021).

Currently, chitin was identified in the skeletons of 21 species of marine sponges and three species of freshwater sponges (see Talevski et al., 2020). In particular, Verongiida (Porifera) has been identified as a promising candidate for bioactive compound extraction. A properly prepared, i.e., after demineralization and decellularization processes, the chitinous scaffold of *Aplysina fistularis* has the appropriate size, shape, and porosity for cell culture (Machałowski et al., 2021a). A combination of chitin and hydroxyapatite (HAp) has been proposed to enhance the bone-forming potential of chitin (Kim et al., 2013). HAp shows good integration with hard and soft tissues. It is characterized by osteoinductive, osteoconductive, osteointegrating, bioaffinity, biocompatibility, and bioactivity properties. HAp promotes osteoblastic cell adhesion, growth, and differentiation, resulting in the deposition of new bones as a replacement of living bones. Despite all its advantages, HAp has low mechanical strength. Therefore, it is advisable to combine it with a material with good mechanical stiffness to improve its properties (Machałowski et al., 2022).

An interaction between DPSCs and a chitosan scaffold as well as a hydroxyapatite scaffold has been reported. DPSCs were able to survive and differentiate in chitosan scaffolds which provided a favorable microenvironment conducive to their survival and differentiation (Zhang et al., 2016). Calcium phosphate-based scaffolds supported the adhesion, proliferation, and differentiation of DPSCs (Khojasteh et al., 2015). However, the comparison of adhesion, proliferation, and ability to differentiate into osteoblasts on the natural chitinous scaffold of *Aplysina fistularis* and on this type of chitin scaffold modified with hydroxyapatite has never been investigated previously. In this work, we present, for the first time, the biological responses such as proliferation, adhesion, and differentiation of human DPSCs on the purified 3D chitinous scaffold from the marine demosponge *Aplysina fistularis* and on the purified 3D chitinous scaffold modified by hydroxyapatite.

## 2 Materials and methods

### 2.1 Scaffold

#### 2.1.1 Chitinous scaffold isolation from *A. fistularis* marine sponge

Air-dried samples of *Aplysina fistularis* marine sponge were purchased from a scientific sponge aquaculture in the Adriatic Sea (Kotor Bay, Montenegro) and delivered by the Internationales Institut für Biomineralogie GmbH (INTIB GmbH, Freiberg, Germany).

The chitinous scaffold was obtained by the standard chemical isolation method, in a treatment consisting of three basic steps (for details, see Machałowski et al., 2021a).

The isolation processes began with immersion of the collected sponge fragments in deionized water for 4 h to remove the corresponding water-soluble compounds and other impurities (I). The isolated sponge skeleton was then immersed in 2.5 M NaOH for 2 days at 40°C to remove proteins (II). The partially deproteinized skeleton was then neutralized with water and immersed for 5 h in 20% acetic acid (25°C) to remove calcium carbonate residues (III). The aforementioned procedure (II–III) was repeated until transparent, pigment-free chitin was obtained (approximately 7 days). Finally, the purified chitin was stored in 70% ethanol at 4°C for further processing.

#### 2.1.2 Synthesis of chitin–HA material

The synthesis of hydroxyapatite/chitin biocomposites was performed by the modified precipitation method, as described previously (Machałowski et al., 2021a). Two solutions (1:1 v/v 50 mL each) were prepared: (I) 0.1 M calcium chloride dihydrate (CaCl_2_∙2H_2_O) (Sigma-Aldrich, St. Louis, MO, USA) + 0.4 M sodium citrate tribasic dihydrate (Na_3_(Cit)∙2H_2_O) (Sigma-Aldrich) + isolated chitin scaffold (fragment 10 cm × 10 cm) and (II) 0.12 M sodium phosphate dibasic (NaHPO_4_) (Chempur, Poland) were transferred to a hydrothermal reactor (Hydrion Scientific, Baltimore, MD, United States). The pH of the mixture was adjusted to 8.5 with 0.1 M NaOH. The mixture was then stored at 80°C for 24 h. To remove any unbound particles of HAp, the obtained biocomposite was cleaned in an ultrasonic bath (Elmasonic GmbH, Singen, Germany) for 1 h and stored at 4°C in 70% ethanol mixture for further analysis. Reference HAp particles were synthesized in an analogous manner but without the addition of chitin in the first step.

#### 2.1.3 Digital microscopy

The prepared scaffolds were visualized using an advanced digital microscope set consisting of a VHX-7000 microscope (Keyence, Japan) and swing head zoom lenses VH-Z20R (magnification up to ×200). The observations were performed in a hydrated state (immersed under water) to visualize their natural morphology.

#### 2.1.4 Thermogravimetric analysis

The thermal stability of the samples was determined by thermogravimetric analysis (TGA) using the Jupiter STA 449 F3 analyzer (Netzsch, Germany). In each measurement, 10 mg of the sample were placed on the analyzer using an Al_2_O_3_ thermobalance. The analysis was carried out at a heating rate of 10°C/min in a nitrogen atmosphere in the temperature range of 25°C–1,000°C.

#### 2.1.5 ATR-FTIR spectroscopy

The ATR FTIR infrared spectroscopy technique (attenuated total reflectance) was used for the characterization of the scaffolds. Infrared spectra were recorded using a VERTEX 70 spectrometer (Bruker, Karlsruhe, Germany). A wide wavenumber range of 4,000–400 cm^−1^ was recorded with a resolution of 0.5 cm^–1^. Scaffold fragments measuring 5 mm × 5 mm and the pure HAp powder (20 mg) were used for the analysis.

#### 2.1.6 Scanning electron microscopy with energy-dispersive X-ray analysis

The scaffolds were immersed in a mixture of 2.5% glutaraldehyde (SERVA Electrophoresis, Heidelberg, Germany) and cacodylate buffer (0.2 M, pH 7.4, SERVA Electrophoresis) for 1 h. Subsequently, the scaffolds were rinsed with cacodylate buffer three times (5 min each, at room temperature) and fixed in 1% osmium tetroxide in cacodylate buffer (SERVA Electrophoresis) for 1 h at 4°C. Afterward, the scaffolds were washed three times with cacodylate buffer (5 min each). The samples were dehydrated in increasing concentrations of ethanol (50%, 70%, 80%, and 96%; Stanlab, Lublin, Poland) for 15 min at 4°C in each solution, followed by incubation in absolute ethanol (three times, 15 min, RT). Afterward, the samples were washed with pure acetone, air-dried, and covered with 30 nm of gold using a high-vacuum sputter coater (Edwards, Burgess Hill, United Kingdom). The JEOL JSM-6610A scanning electron microscope, operated at 20 kV, with a secondary electron detector, was used for observation and imaging. Moreover, a JEOL JEE 4B vacuum evaporator was used. The distributions of element concentrations were obtained in the form of EDX pattern maps.

### 2.2 Cell lines

Human DPSCs (Lonza, Basel, Switzerland) were isolated from adult third molars harvested during the extraction of a donor’s wisdom teeth. DPSCs were cryopreserved at the primary passage, and the DPSC Dental Pulp Stem Cell BulletKit™ Medium (Lonza) was optimized for cell maintenance and expansion. DPSCs showed MSC characteristics according to the surface protein expression profile and expressed CD105, CD166, CD29, CD90, and CD73 and did not express CD34, CD45, and CD133, according to the manufacturer’s specifications. DPSCs were cultured in the DPSC Dental Pulp Stem Cell BulletKit™ Medium (Lonza), according to the manufacturer’s instructions.

The human normal osteoblast cell line, hFOB 1.19 (ATCC, Old Town Manassas, VA, USA), was cultured in a DMEM:F12 medium supplemented with 10% fetal calf serum and 1% of L-glutamine with a penicillin and streptomycin solution (Sigma-Aldrich, St. Louis, MO, United States). Media were changed twice weekly, cells were passed at approximately 70% confluence, and cultures were maintained under standard conditions: 37°C, 5% CO_2_, and a humid atmosphere.

### 2.3 Cell culture on scaffolds

DPSCs and hFOB 1.19 cells (1.0 × 10^5^) were seeded in pure chitin scaffolds (PURE) and also in the chitin scaffold modified with hydroxyapatite (HAp) measuring 0.5 cm × 0.5 cm × 0.4 cm placed in a six-well plate (TPP, Trasadingen, Switzerland). Cell cultures on the scaffolds were performed with an appropriate complete medium for 5 and 10 days, and there were two points of differentiation analyzed in the study. Gentle rocking was used to achieve real adhesion of the cells to the surface of the chitin scaffolds (Zak et al., 2023). The cells and scaffolds with cells were collected for the following assays.

### 2.4 Staining methods

#### 2.4.1 Alizarin S staining

Alizarin S, an anthraquinone dye, was used to assess calcium deposition in cells. The culture medium and cells were removed. The scaffolds containing cells were gently washed three times with PBS. The cells were then fixed in 4% formaldehyde (Chempur^®^, Piekary Slaskie, Poland) for 15 min at room temperature and washed three times with diH_2_O. After this step, diH_2_O was removed, and 2 mL of 40 mM Alizarin S staining solution (cat. 8678, ScienCell Research Laboratories, Carlsbad, CA, United States) was added to each well. After 30 min of incubation at room temperature, the scaffolds were washed five times in diH_2_O, and the cells were observed under an inverted phase-contrast microscope (CKX53, Olympus, Tokyo, Japan).

#### 2.4.2 Alkaline phosphatase staining

The expression of alkaline phosphatase was visualized with the Alkaline Phosphatase Staining Kit (Purple) (ab242287, Abcam, Cambridge, United Kingdom). After removal of the culture medium, the scaffolds were washed with PBS/0.1% Tween buffer and incubated in a fixing solution from the kit for 2 min at room temperature. The scaffolds were then washed PBS/0.1% Tween buffer and incubated with an ALP staining solution for 30 min at room temperature, protected from direct sunlight. The ALP staining was then removed, the scaffolds were rinsed in PBS, and the cells were observed under the inverted phase-contrast microscope (CKX53, Olympus).

#### 2.4.3 Neutral red staining

Neutral red uptake (0.33% solution, cat. N2889, Sigma-Aldrich) was used to visualize live cells on the scaffold surface. For each scaffold, 12 µL of neutral red solution was added per 1 mL of the medium and incubated for 2 h at 37°C. Afterward, cells on the scaffold surface were observed under the inverted phase-contrast microscope (Olympus, CKX53) and an Eclipse 80i fluorescence microscope (Nikon Corporation, Tokyo, Japan).

#### 2.4.4 Crystal violet staining

Fixed cells were washed twice with cold PBS (Lonza) and then incubated for 10 min in cold methanol (−20°C) (Chempur^®^). The cells were then stained for 10 min in 0.5% crystal violet solution in 25% methanol (Sigma-Aldrich^®^). The cells with crystal violet staining were then washed several times in water, and the cells on the scaffold surface were observed under an inverted phase-contrast microscope (Olympus, CKX53).

### 2.5 Scanning electron microscopy

The scaffolds were fixed in 2.5% glutaraldehyde (SERVA Electrophoresis, Heidelberg, Germany) diluted in cacodylate buffer (0.2 M, pH 7.4, SERVA Electrophoresis) for 1 h. Next, the scaffolds with cells were washed three times in cacodylate buffer (5 min, RT) and fixed in 1% osmium tetroxide in cacodylate buffer (SERVA Electrophoresis) (1 h at 4°C). Afterward, the scaffolds were incubated three times with cacodylate buffer (5 min). The samples were dehydrated using increasing concentrations of ethanol (50%, 70%, 80%, and 96%; Stanlab, Lublin, Poland) for 15 min in each solution at 4°C, and then the samples were incubated in absolute ethanol (three times, 15 min, RT). The samples were then washed in pure acetone, air-dried, and coated with 30 nm of gold in a high-vacuum sputter coater (Edwards, Burgess Hill, United Kingdom). A JSM-6610A scanning electron microscope (JEOL, Tokyo, Japan) with a 20 kV accelerating voltage and a secondary electron detector was used for observation and imaging.

### 2.6 Transmission electron microscopy

The ultrastructure of the hFOB 1.19 cell lines and DPSCs was assessed via transmission electron microscopy (TEM). All cells cultured on scaffolds were fixed in 3.6% glutaraldehyde and processed according to standard procedures (John, 2014).

The modified protocol was applied for the hFOB 1.19 cells and DPSCs cultured without scaffolds. After the fixation and wash steps of samples, droplets of bovine thrombin (Biomed-Lublin, Poland, 1 amp. with 400 a.u. lyophilized dissolved in 5 mL of PBS) and fibrinogen (1 mg/mL; Merck KGaA, Darmstadt, Germany) were used to entrap cells within the fibrin clot. A detailed procedure was described elsewhere (Solarska-Ściuk, K. et al., 2022). The same protocol, reagents, and devices were used in the current study. Electronograms were acquired at magnifications of ×3,000 to ×100,000 using the image platform iTEM1233 provided with a Morada camera (Olympus, Münster, Germany).

### 2.7 Droplet digital PCR

Total RNA was isolated using the RNeasy Mini Kit (QIAGEN, Hilden, Germany). Reverse transcription was performed using iScript™ Reverse Transcription Supermix for RT-qPCR (Bio-Rad, Hercules, CA, USA). An input of 50 ng of RNA from each sample was reverse-transcribed using a C1000 Touch thermal cycler (Bio-Rad). The reactions conditions are as follows: priming for 5 min at 25°C, reverse transcription for 20 min at 46°C, and final reverse transcriptase inactivation for 1 min at 95°C. The droplet digital PCR (ddPCR) reaction mixtures contained 10 µL of 2X ddPCRTM MasterMix for Probes (Bio-Rad), 7.67 µL of molecular biology-grade water, 2.5 µL of RT product, and 1 µL of TaqMan specific probe (Hs00959010_m1 for SPP1, Hs01047973_m1 for RUNX2, and Hs01029144_m1for ALPL) (Applied Biosystems, Foster City, CA, United States). Next, the 20 µL of the reaction mixtures with 50 µL of Droplet Generation Oil for Probes (Bio-Rad) were mixed in the QX100 Droplet Generator (Bio-Rad). Droplets obtained from each sample were then transferred to a 96-well PCR plate (Bio-Rad). PCR amplifications were performed on a C1000 Touch thermal cycler (Bio-Rad). The reaction conditions were as follows: enzyme activation for 10 min at 95°C, followed by 40 cycles of denaturation (for 30 s at 94°C) and annealing/extension (for 1 min at 60°C), and, finally, enzyme deactivation for 10 min at 98°C and 10 min ending at room temperature (RT). The reading of the droplets was then performed automatically by the droplet reader (Bio-Rad). The absolute quantification (AQ) of each miRNA was calculated from the number of positive counts per panel using the Poisson distribution. Results are represented as the mean of three independent replicates. The quantification of the target mRNA is presented as the number of copies/μL in the PCR mixture.

### 2.8 Statistical analysis

Statistical analysis of ddPCR results was performed using Prism 9.3.1 (GraphPad, La Jolla, CA, USA). The normality of distribution was analyzed using the Shapiro–Wilk test. The distribution of variables was normal; therefore, parametric tests were used for statistical analysis. One-way ANOVA with Tukey’s *post hoc* tests for multiple comparisons was used to determine differences between groups. Statistical significance was defined at a level of *p* < 0.05.

## 3 Results

### 3.1 Scaffold characterization

**FIGURE 1:**
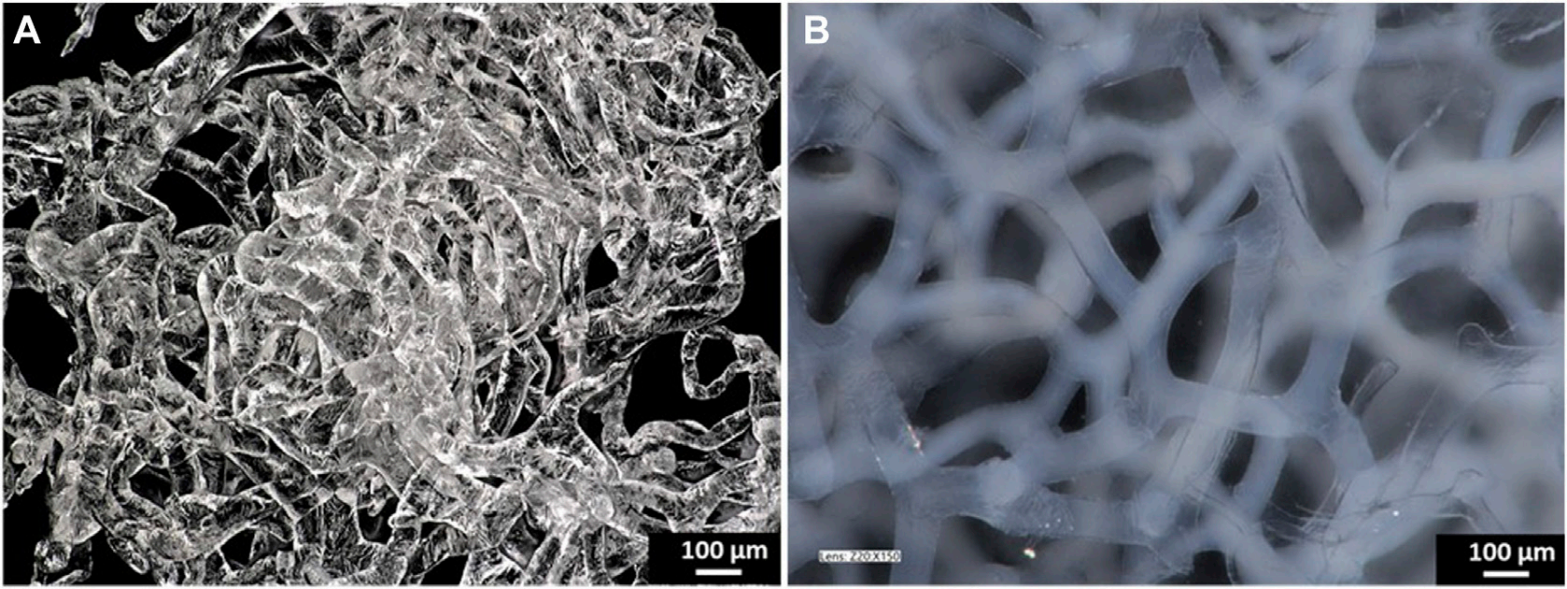
Digital microscopy images of the pure chitinous scaffold of the sponge skeleton of *A. fistularis* after isolation **(A)** and the HAp-modified scaffold in the hydrated state **(B)**.

**FIGURE 2:**
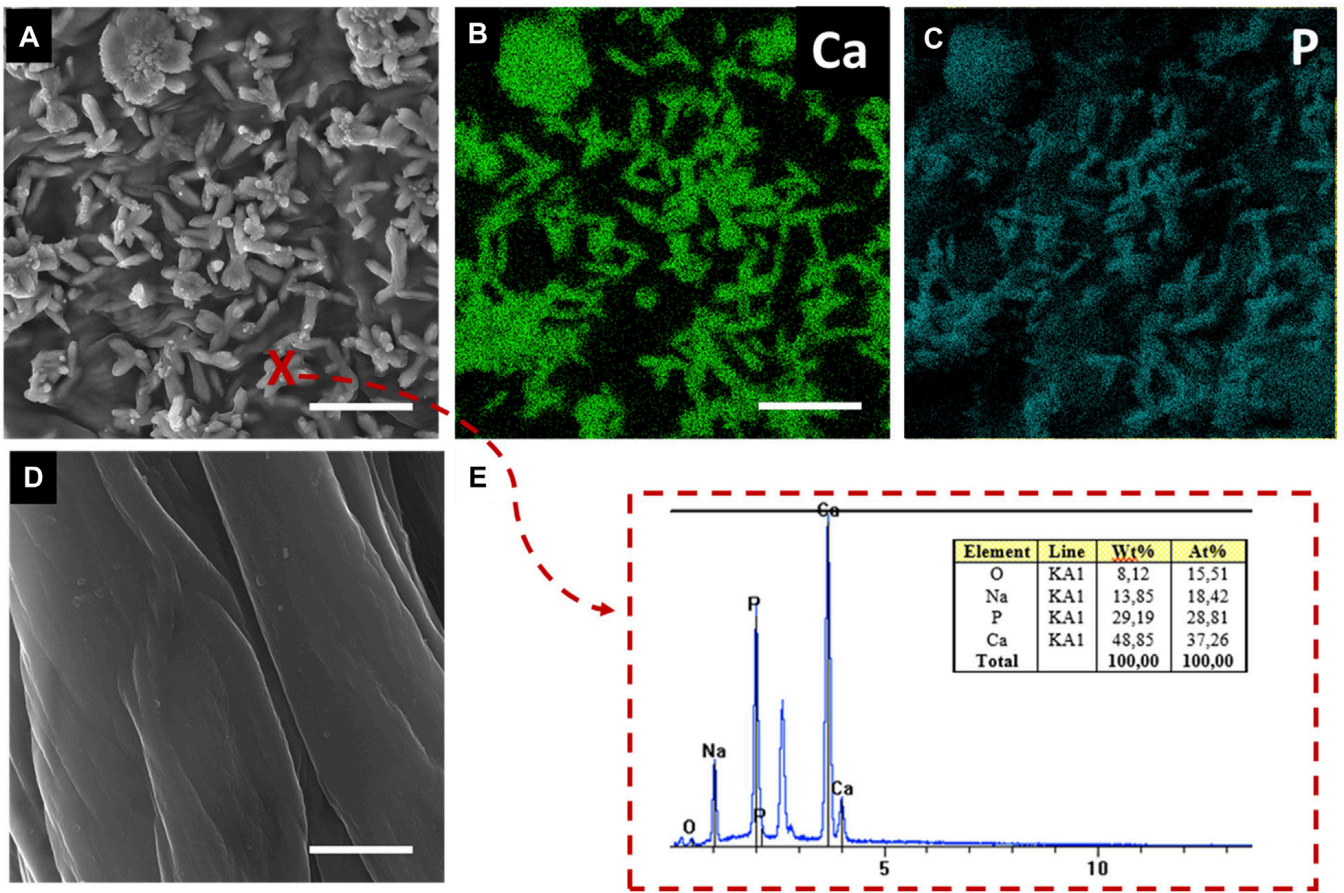
SEM images of the surface of chitinous scaffold fibers functionalized by HAp **(A,E)** and reference pure chitin before functionalization **(D)**. EDX mapping for calcium and phosphate at the scaffold surface **(B,C)**. EDX spectrum of the inorganic phase at the functionalized scaffold surface at point (x). Scale bar = 1 µm.

Figure 1 shows pure and calcium phosphate-modified scaffold obtained from *A. fistularis* marine sponge. As can be observed, a standard 7-day treatment with an alkali–acid-based solution resulted in the isolation of a naturally formed, transparent, cell-free scaffold. The unique interconnected 3D construct constitutes an excellent environment for human cell cultures, as shown previously (Mutsenko et al., 2017; Machałowski et al., 2021a; Machałowski et al., 2022). After the hydrothermal modification of chitin, a clearly visible color change was observed. The scaffold turned white due to the presence of hydroxyapatite particles inside the fibers as well as on their surface. Our digital microscopy observations (Figure 1) were confirmed via scanning electron microscopy (SEM) + energydispersive X-ray (EDX) mapping analysis (Figures 2A–E). Well-visible crystallites of calcium phosphates in the size range of 0.1–1 µm (Figure 2A) were present on the surface of the chitinous scaffold. In contrast, chitin before HAp modification showed a smooth surface without any impurities. The distribution of the chemical elements obtained using EDX analysis proved the presence of calcium phosphates with the Ca/P ratio of 1.67, which is comparable to the hydroxyapatite in the mature human bone (Szatkowski, T. et al., 2015; Mahamid, J. et al. (2011).

**FIGURE 3:**
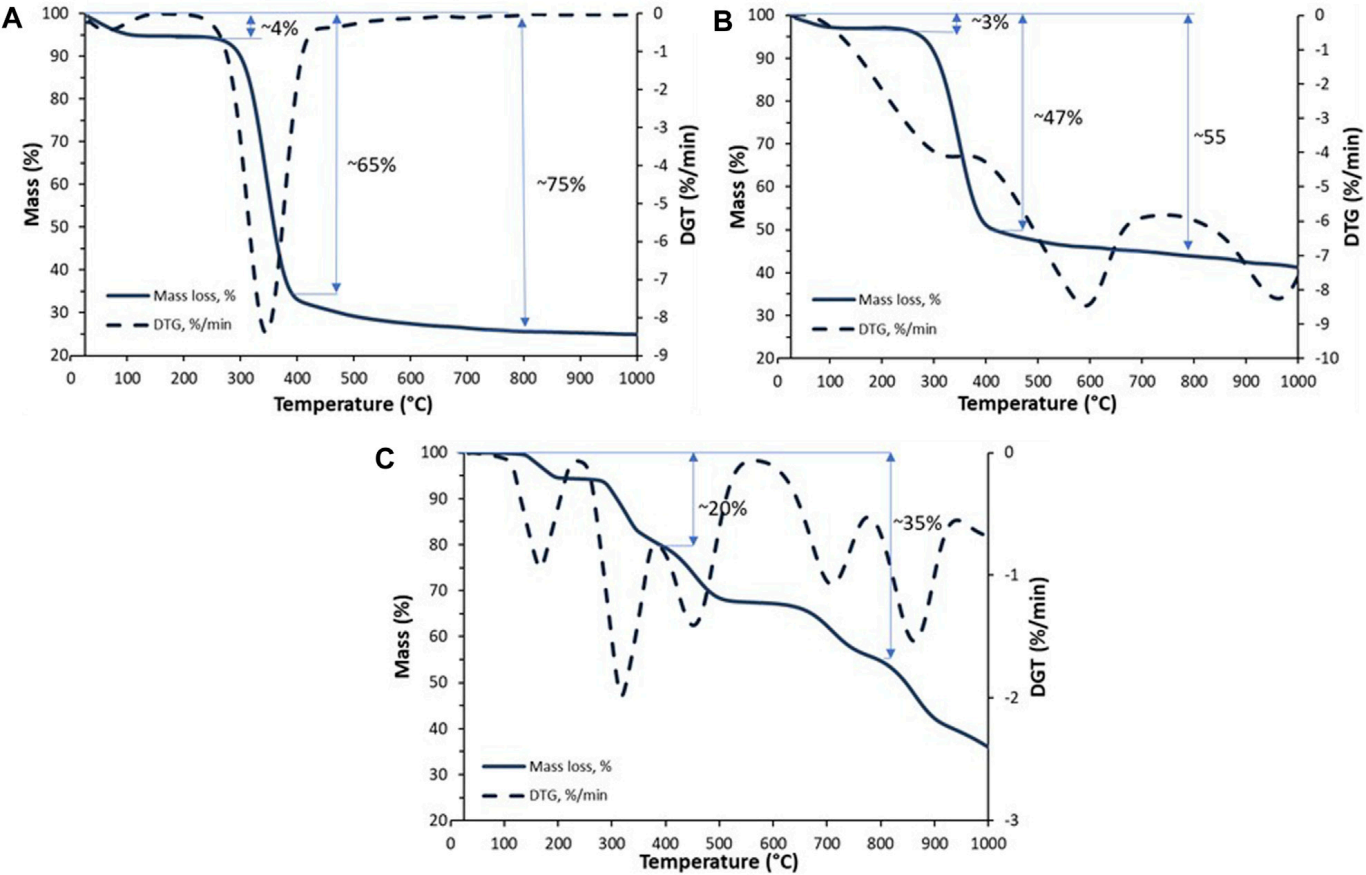
TGA/TTG curves of the pure chitinous scaffold of *A. fistularis* **(A)**, scaffold modified with HAp **(B)**, and the pure HAp powder **(C)**.

The graphs in Figure 3 show the thermal stability of the materials obtained. First, the parameters of the pure chitin scaffold were developed. Two significant decreases were observed during the analysis. As shown in Figure 3A, in the temperature range of 100°C–120°C, the loss of physically and chemically bound water was detected due to the previous preparation (Szatkowski et al., 2015) of the sample. Due to the previous sample preparation (air-drying), the observed decrease was measured as only 4%. Subsequently, a significant mass loss with a maximum rate of 8%/min was observed in the temperature range of 200°C–400°C. These data confirmed thermal and oxidative decomposition of chitin (Kaya et al., 2017). Further analysis was performed for the scaffold modified by hydroxyapatite. As observed, the prepared material showed higher thermal stability at 350°C, with an estimated mass loss of 47% (compared to pure chitin—65%). Without any doubt, this is due to the presence of an inorganic phase (Tõnsuaadu, 2012). Dehydroxylation of hydroxyapatite is a process that starts in the temperature range of 800°C–900°C. (Tõnsuaadu, 2012). This process is followed by the formation of vacancies in the position of the hydroxyl groups in the structure of HAp and could be recorded as a small weight loss (Figures 3B, C). Therefore, these curve points can be used to calculate the estimated inorganic phase content. As shown, the difference between the masses of pure and modified scaffolds is close to 20%.

**FIGURE 4:**
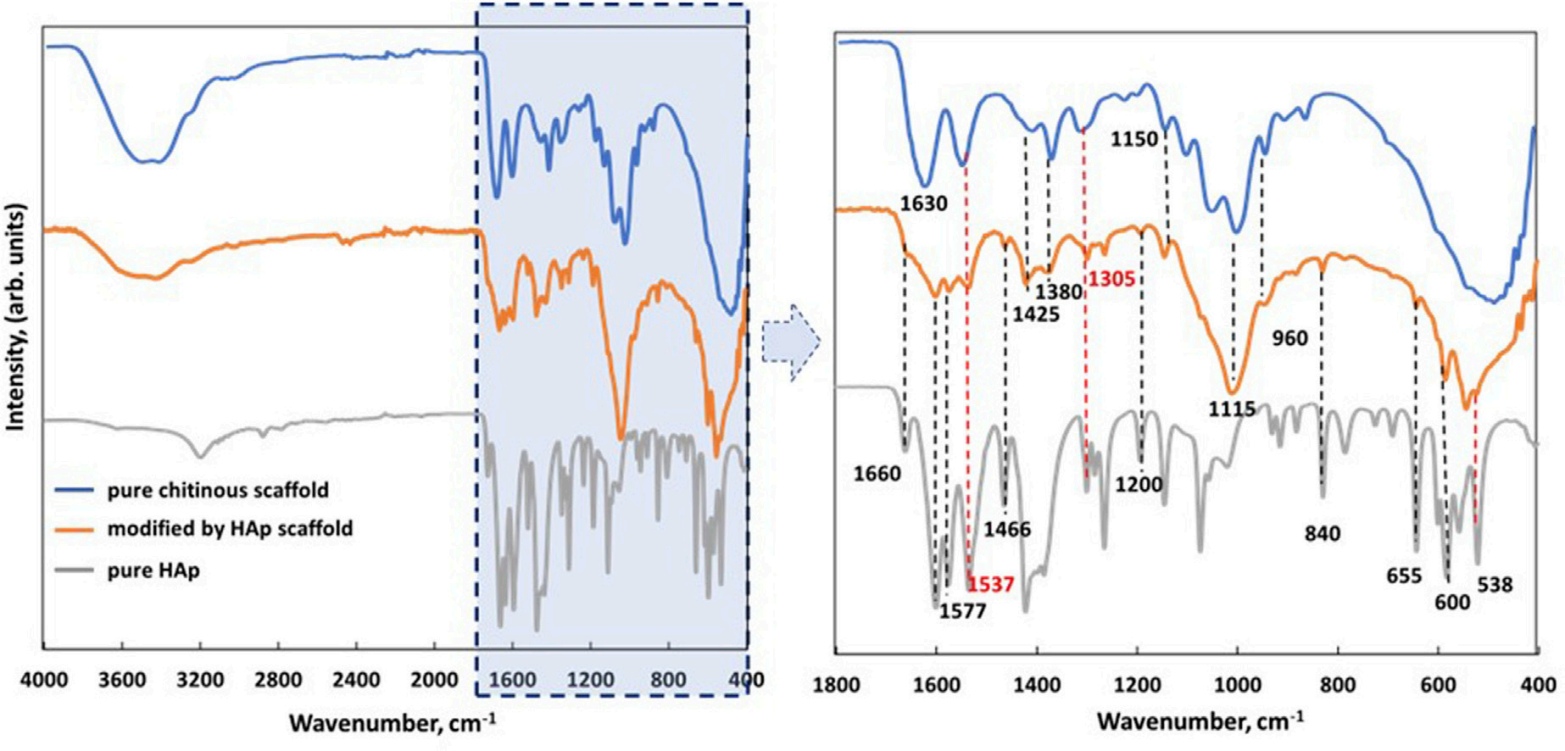
FTIR spectra of isolated pure chitinous scaffolds from the sponge skeleton of *A. fistularis* (blue), scaffold modified with HAp (orange), and pure HAp obtained during synthesis as a reference (gray).

FTIR analysis was used to visualize the interactions between chitin (organic) and HAp (inorganic) in the prepared biocomposite. Figure 4 shows the spectra of the pure chitinous scaffold (blue), the chitinous scaffold modified with HAp (orange), and the pure HAp powder unattached to the scaffold during synthesis (gray). The degree of acetylation (DA) of isolated chitin was estimated as 71% and the degree of deacetylation (DD) as 29%. These data are in agreement with previously obtained DA and DD observed for alkali-based isolated chitin. Gbenebor et al. (2017) described similar values for alkali-treated chitin isolated from shrimps’ skeletons.

Comparing the modified scaffold with pure chitin, the peaks of stretching vibrations from both inter- (C=O⋅⋅⋅H–N) and intramolecular (C=O⋅⋅⋅HOCH_2_) hydrogen bonds (amide I—1,630 cm^−1^) overlapped with the peak of OH stretching vibration, which suggests that the hydrogen bonds of chitin chains were wakened by HAp particles. However, several of the characteristic bands of chitin can be distinguished in a HAp-modified scaffold, for example, the band at 1,425 cm^−1^, corresponding to CH_2_ stretching vibrations of chitin (Kumirska et al., 2010). Another band was detected at 1,375 cm^−1^, corresponding to the asymmetric deformation of CH_3_ of the chitin chain (Kumirska et al., 2010). In particular, an interesting signal was observed at 1,305 cm^−1^ (amide III—νC–N and δN–H), which proved the presence of α-chitin in the analyzed sample (Nowacki et al., 2020). In the spectrum of the obtained biocomposite, signals of HAp can also be distinguished. As previously described, signals at 960, 655, and 603 cm^−1^ should be attributed as fingerprints to the phosphate groups (Chang et al., 2013). Moreover, the presence of HAp was confirmed by XRD analysis (see Supplementary Material).

### 3.2 Cell cultures on scaffolds

**FIGURE 5:**
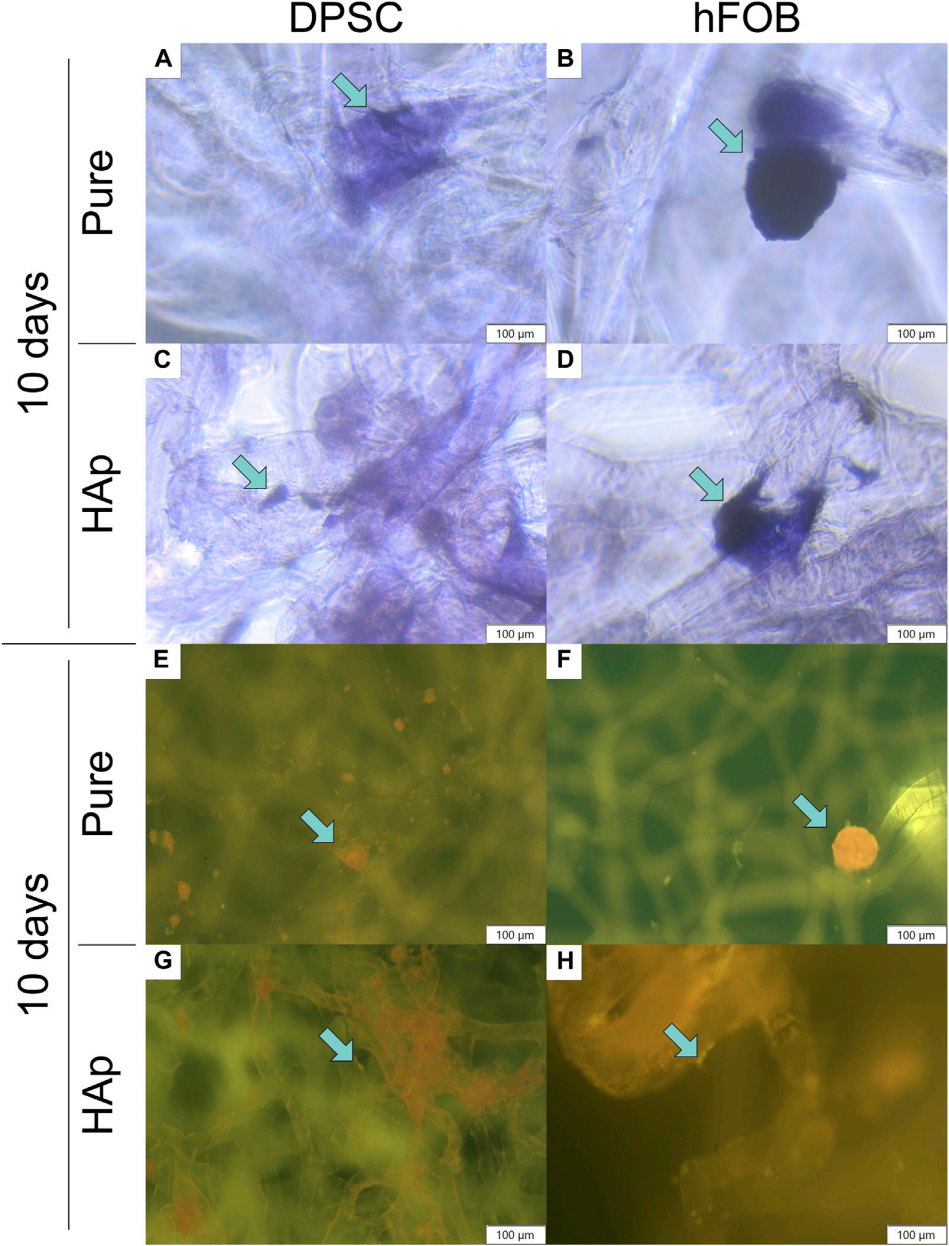
Crystal violet and neutral red staining after 10 days of cell incubation. Crystal violet staining: **(A)** DPSCs cultured on the pure chitin scaffold (PURE). **(B)** hFOB 1.19 cells cultured on the pure chitin scaffold (PURE). **(C)** DPSCs cultured on the chitin scaffold modified with hydroxyapatite (HAp). **(D)** hFOB 1.19 cells cultured on the chitin scaffold modified with hydroxyapatite (HAp). Neutral red staining (fluorescence microscopy): **(E)** DPSCs cultured on the pure chitin scaffold (PURE). **(F)** hFOB 1.19 cells cultured on the pure chitin scaffold (PURE). **(G)** DPSCs cultured on the hydroxyapatite-modified (HAp) chitin scaffold. **(H)** hFOB 1.19 cells cultured on the chitin scaffold modified with hydroxyapatite (HAp). Blue arrows indicate cells colonizing the scaffold surface. Magnification: ×40.

Our results showed single cells of DPSCs and hFOB 1.19 after 24 h of incubation. After 5 days of investigation, clusters of the cells were visible on the surface of pure scaffolds (PURE) and also on chitin scaffolds modified with hydroxyapatite (HAp). hFOB 1.19 clusters exhibited a round morphology, while the DPSC clusters coated both scaffolds (Figure 5). Detailed colony formation and osteogenic or odontogenic differentiation of DPSCs and hFOB 1.19 on scaffolds were assessed via SEM, TEM, and the following investigation methods.

### 3.3 Staining methods

Neutral red and crystal violet staining methods were performed to confirm cell adhesion to the scaffolds, as shown in Figure 5. The analysis of crystal violet staining after 5 and 10 days of incubation revealed that DPSCs and hFOB 1.19 cells were attached to the surface of the pure scaffold as well as the scaffold modified with HAp. An increased number of cells on the surface of the scaffolds were observed after 10 days of culture (see Figures 5A–D).

**FIGURE 6:**
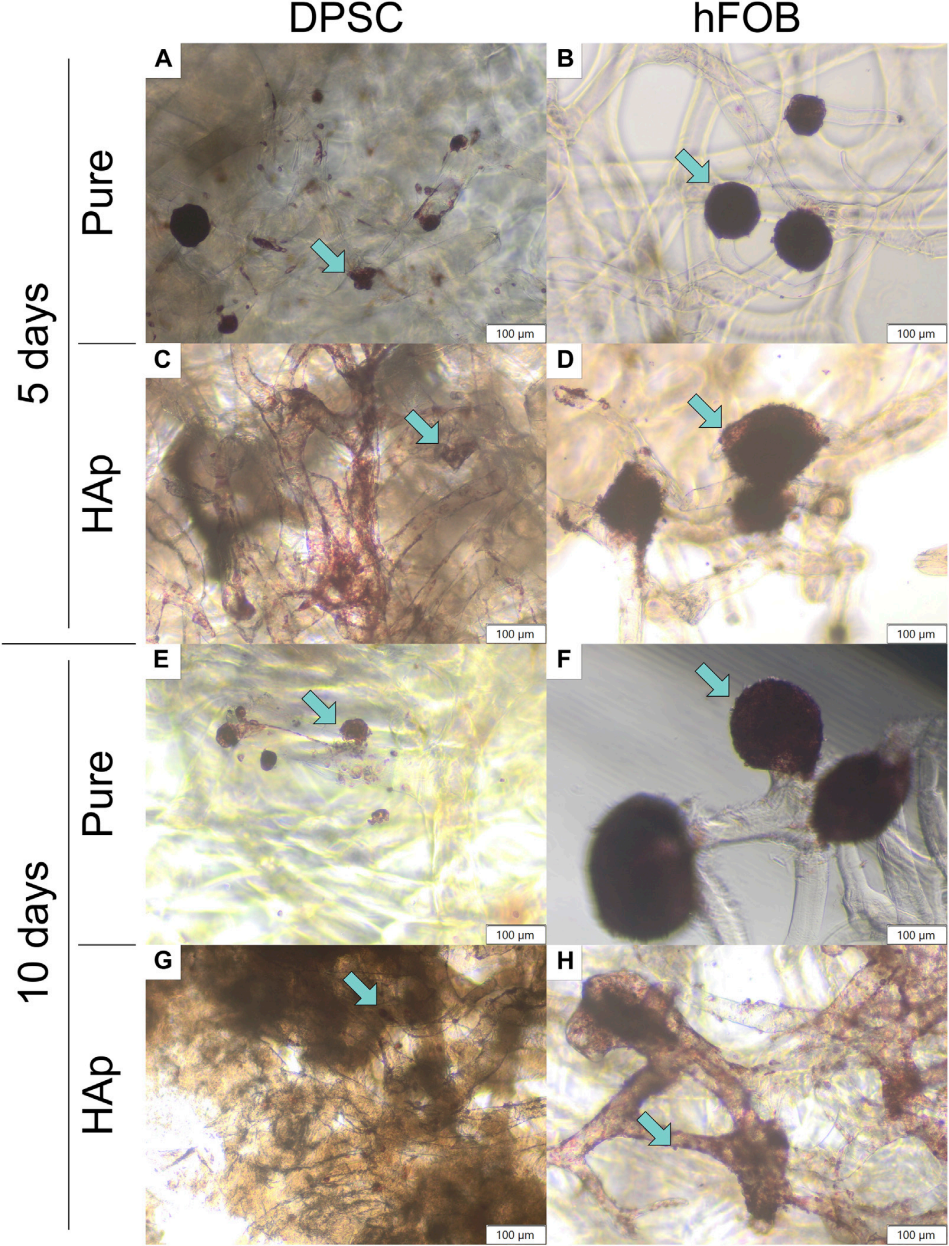
Neutral red staining after 5 and 10 days of cell incubation. **(A)** DPSCs cultured on the pure chitin scaffold (PURE) after 5 days. **(B)** hFOB 1.19 cells cultured on the pure chitin scaffold (PURE) after 5 days. **(C)** DPSCs cultured on the chitin scaffold modified with hydroxyapatite (HAp) after 5 days. **(D)** hFOB 1.19 cells cultured on the chitin scaffold modified with hydroxyapatite (HAp) after 5 days. **(E)** DPSCs cultured on the pure chitin scaffold (PURE) after 10 days. **(F)** hFOB 1.19 cells cultured on the pure chitin scaffold (PURE) after 10 days. **(G)** DPSCs cultured on the chitin scaffold modified with hydroxyapatite (HAp) after 10 days. **(H)** hFOB 1.19 cells cultured on the chitin scaffold modified with hydroxyapatite (HAp) after 10 days. Blue arrows indicate cells colonizing the scaffold surface. Magnification: ×40.

Biocompatibility tests were performed to verify the viability of DPSCs in contact with chitin scaffolds and hydroxyapatite-modified scaffolds using neutral red staining, as shown in Figures 5E–H and in Figure 6. Our results indicate the presence and spatial distribution of viable DPSCs on both types of scaffolds after 5 and 10 days of incubation. A similar distribution of DPSCs has been observed on pure chitin scaffolds and on chitin scaffolds modified with hydroxyapatite. Cell adhesion to both types of scaffolds was also verified via SEM. Our studies demonstrate that DPSCs and hFOB 1.19 cells adhere and proliferate on the scaffolds.

**FIGURE 7:**
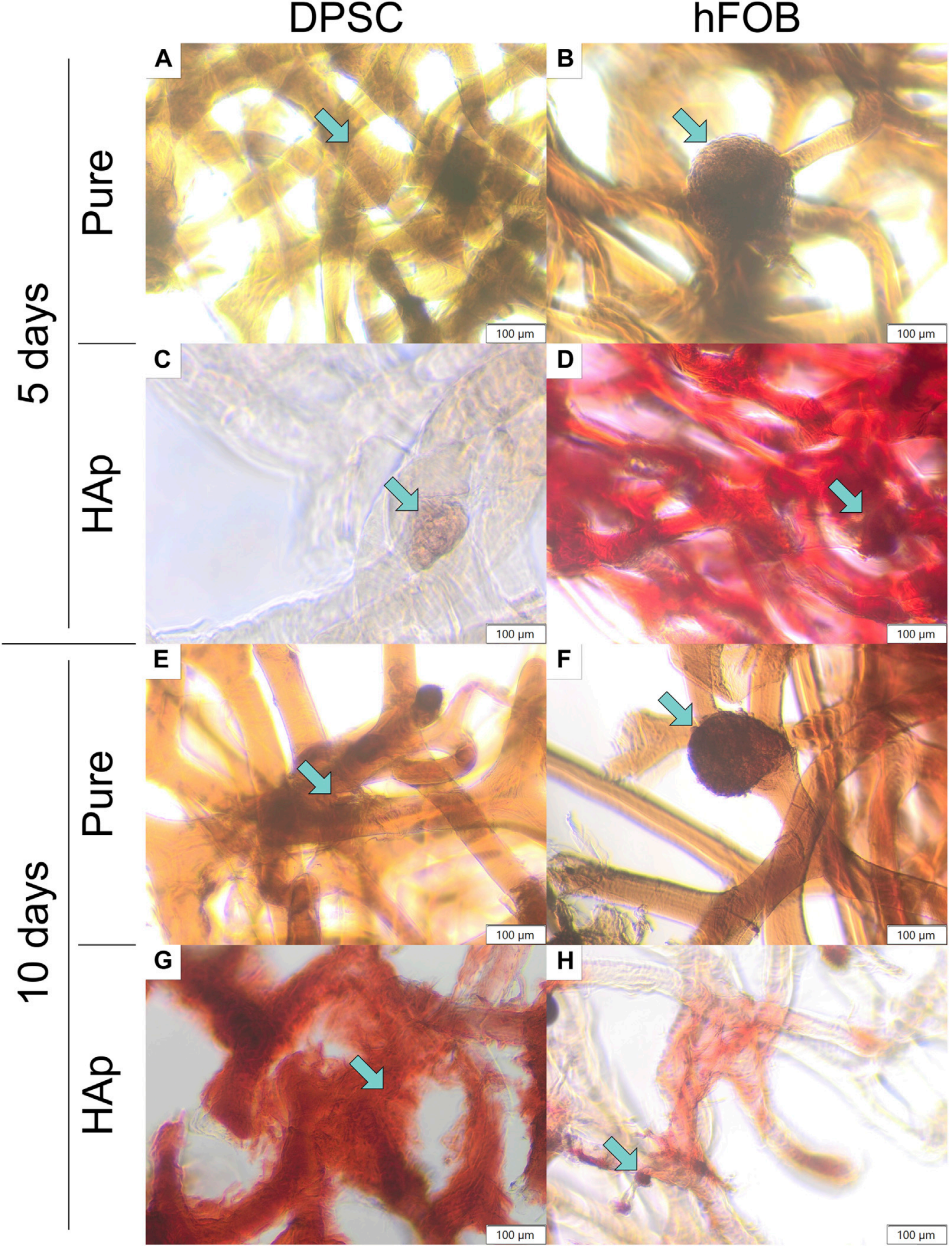
Alizarin Red S staining after 5 and 10 days of cell incubation. **(A)** DPSCs cultured on the pure chitin scaffold (PURE) after 5 days. **(B)** hFOB 1.19 cells cultured on the pure chitin scaffold (PURE) after 5 days. **(C)** DPSCs cultured on the chitin scaffold modified with hydroxyapatite (HAp) after 5 days. **(D)** hFOB 1.19 cells cultured on the chitin scaffold modified with hydroxyapatite (HAp) after 5 days. **(E)** DPSCs cultured on the pure chitin scaffold (PURE) after 10 days. **(F)** hFOB 1.19 cells cultured on the pure chitin scaffold (PURE) after 10 days. **(G)** DPSCs cultured on the chitin scaffold modified with hydroxyapatite (HAp) after 10 days. **(H)** hFOB 1.19 cells cultured on the chitin scaffold modified with hydroxyapatite (HAp) after 10 days. Blue arrows indicate cells colonizing the scaffold surface.

To determine the deposition in cells, Alizarin Red S staining was performed for 5 and 10 days, as shown in Figure 7. The scaffold cultured cells appeared white before staining, and the scaffold had turned red after staining, as shown in Figure 7. The HAp scaffold showed the highest staining level of Alizarin Red S compared to other samples, confirming that the calcium particles were exposed in cells on the scaffold surface. After 10 days, the color became slightly richer than that after 5 days, and hFOB 1.19 cells appeared redder than DPSCs.

### 3.4 Alkaline phosphatase activity in differentiating stem cells

**FIGURE 8:**
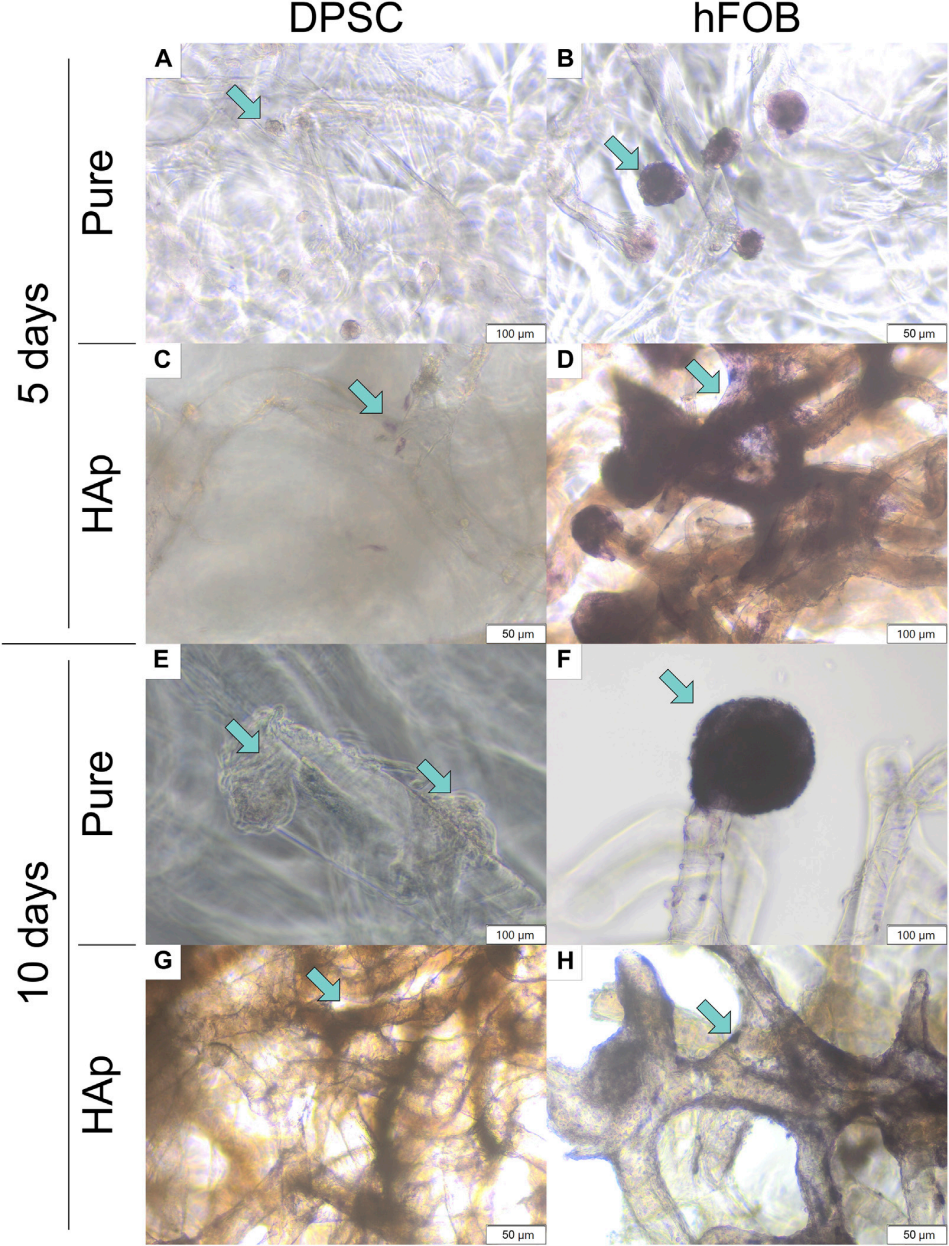
Alkaline phosphatase activity after 5 and 10 days of cell incubation. **(A)** DPSCs cultured on the pure chitin scaffold (PURE) after 5 days. **(B)** hFOB 1.19 cells cultured on the pure chitin scaffold (PURE) after 5 days. **(C)** DPSCs cultured on the chitin scaffold modified with hydroxyapatite (HAp) after 5 days. **(D)** hFOB 1.19 cells cultured on the chitin scaffold modified with hydroxyapatite (HAp) after 5 days. **(E)** DPSCs cultured on the pure chitin scaffold (PURE) after 10 days. **(F)** hFOB 1.19 cells cultured on the pure chitin scaffold (PURE) after 10 days. **(G)** DPSCs cultured on the chitin scaffold modified with hydroxyapatite (HAp) after 10 days. **(H)** hFOB 1.19 cells cultured on the chitin scaffold modified with hydroxyapatite (HAp) after 10 days. Blue arrows indicate cells colonizing the scaffold surface. Magnification: ×40.

An alkaline phosphatase (ALP) assay was performed to determine the early osteogenic differentiation of DPSCs cultured on the pure chitin scaffold (PURE) and on the chitin scaffold modified with hydroxyapatite (HAp). The results are shown in Figure 8.

The study showed that the expression of alkaline phosphatase detected in hFOB 1.19 cells was higher compared to that in DPSCs. After 10 days of culture, a colony of cells coating the scaffolds with alkaline phosphatase was also detected. No differences were observed between the days of culture and the scaffold type, and alkaline phosphatase was typically expressed for osteoblast cells. In the case of DPSC culture on scaffolds, most characteristic changes were observed after 10 days. The colony of cells cultured on a pure chitin scaffold resulted in a lack of alkaline phosphatase, while on HAp scaffolds, a typical staining for alkaline phosphatase was observed (Figure 8). This might suggest a positive effect of hydroxyapatite on the initial step of differentiation to odontoblasts or osteoblasts.

### 3.5 Scanning electron microscopy

**FIGURE 9:**
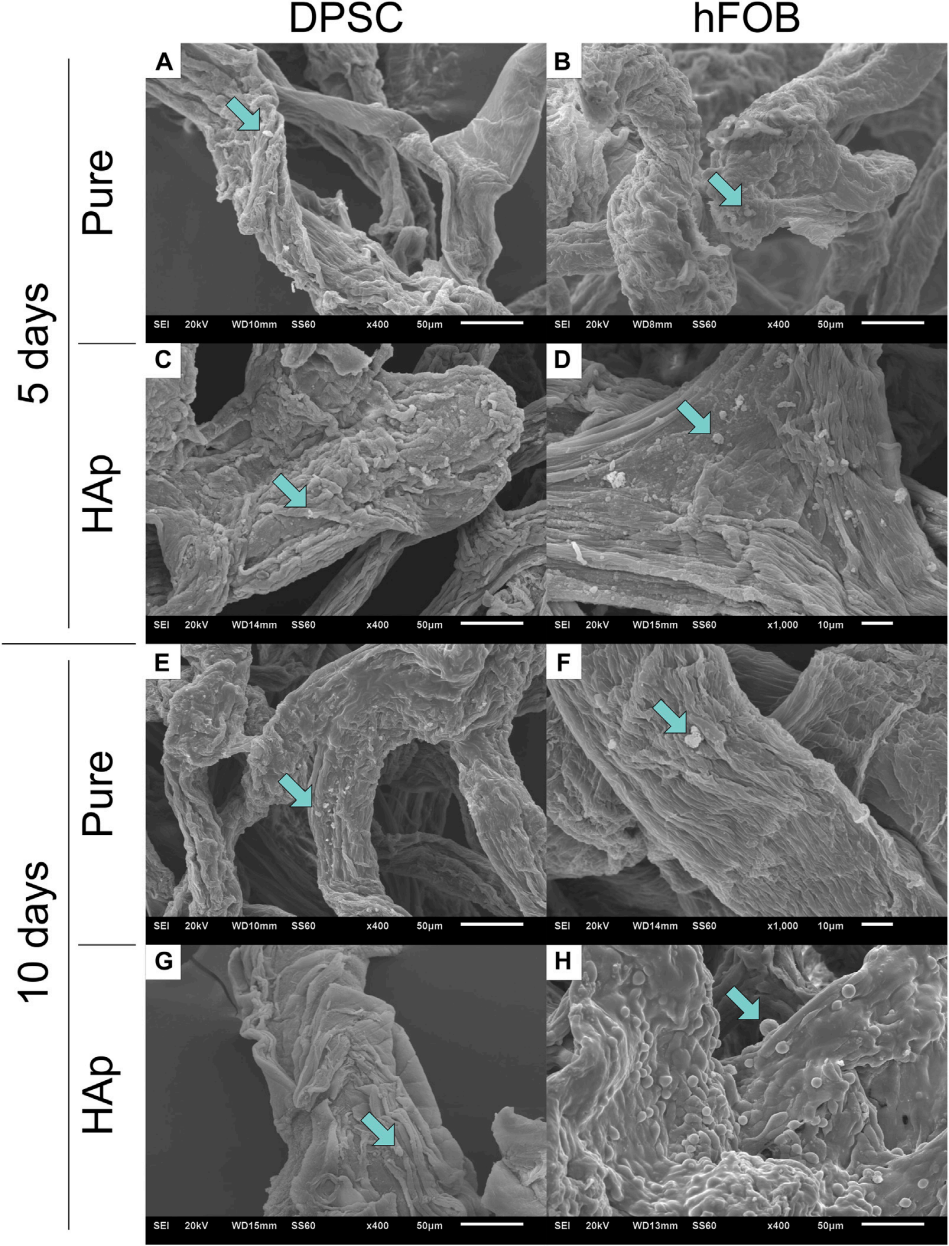
Scanning electron microscopy (SEM) after 5 and 10 days of cell incubation. **(A)** DPSCs cultured on the pure chitin scaffold (PURE) after 5 days. **(B)** hFOB 1.19 cells cultured on the pure chitin scaffold (PURE) after 5 days. **(C)** DPSCs cultured on the chitin scaffold modified with hydroxyapatite (HAp) after 5 days. **(D)** hFOB 1.19 cells cultured on the chitin scaffold modified with hydroxyapatite (HAp) after 5 days. **(E)** DPSCs cultured on the pure chitin scaffold (PURE) after 10 days. **(F)** hFOB 1.19 cells cultured on the pure chitin scaffold (PURE) after 10 days. **(G)** DPSCs cultured on the chitin scaffold modified with hydroxyapatite (HAp) after 10 days. **(H)** hFOB 1.19 cells cultured on the chitin scaffold modified with hydroxyapatite modification (HAp) after 10 days.

The SEM image of the microscopic porous structure of the 3D scaffolds is shown in Figure 9. SEM observations were made after 5 and 10 days of culture. Previous research shows that porosity is important for the mechanical and biological characteristics of the scaffold in bone tissue engineering and plays a crucial role in nutrient and gas exchange (Annabi et al., 2010). Both scaffolds—the pure chitinous scaffold and the scaffold modified with HAp—mimic the organization of the 3D dimensions of tissues allowing for cellular attachment and spreading. DPSCs coat the scaffold surface, penetrate it, and proliferate inside. In particular, dense colonies of DPSCs coated the surface of the HAp-modified scaffold after 10 days. Compared to the control group, hFOB 1.19 cells exhibited round-shaped clusters. hFOB 1.19 cells attached and spread on pure and modified scaffolds. The SEM image presents a progressive increase of hFOB 1.19 cells on the chitin scaffold modified with HAp.

### 3.6 Transmission electron microscopy analysis

**FIGURE 10:**
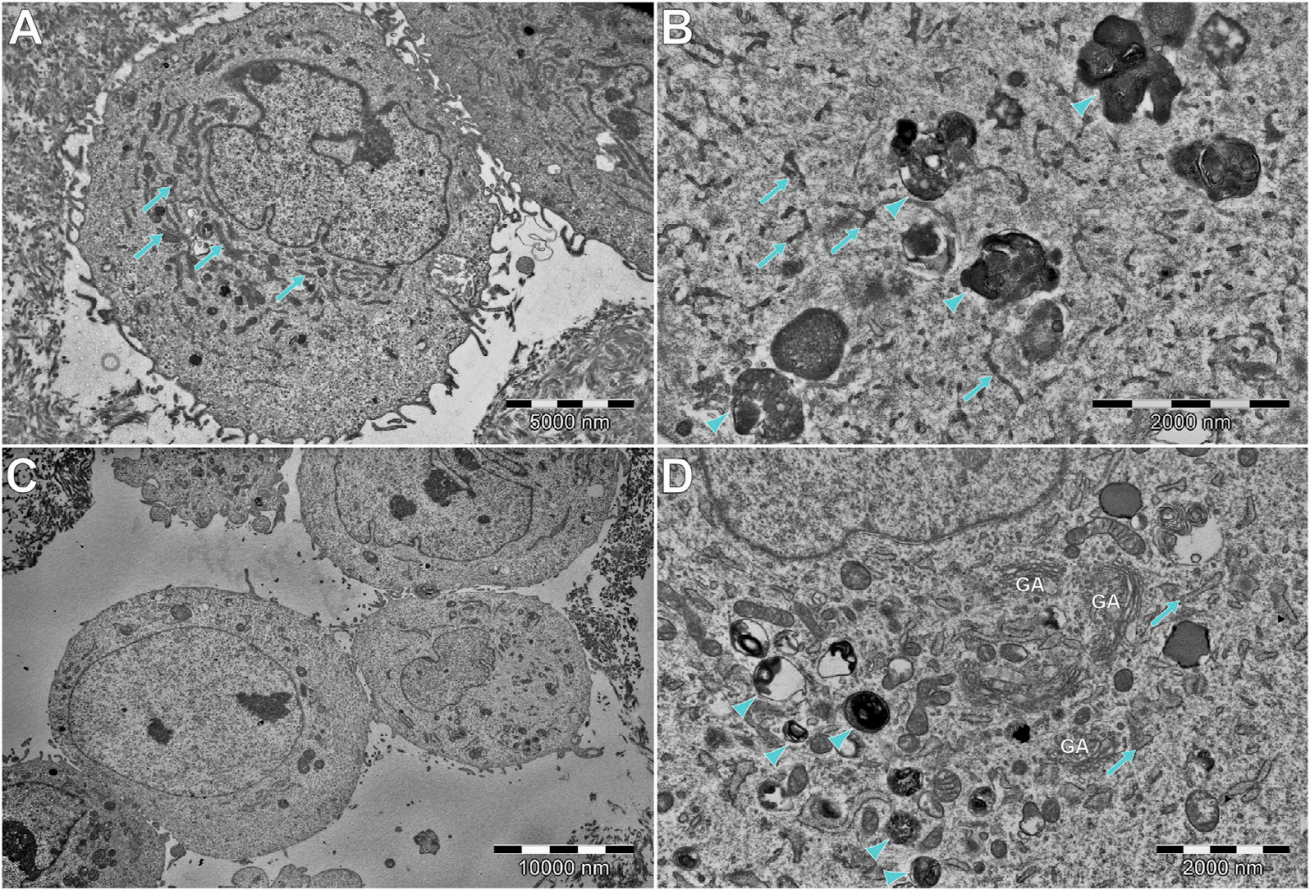
Comparison of **(A,B)** DPSCs and **(C,D)** hFOB cells, cultured without scaffold and HAp. **(A,B)** DPSCs tend to have extensive and narrow rough endoplasmic reticulum (RER; arrows). In some cells, electron-dense multivesicular bodies (arrowheads) can be found. **(C)** Most of the hFOB cells cultured without any scaffold of HAp show a rather modest ultrastructure, without abundant organelles of any type. However, some of them **(D)** show abundant multilamellar bodies (arrowheads), without visible crystallization. Moreover, an extensive number of Golgi apparatus (GA) and wide RER (arrows) can be found.

**FIGURE 11:**
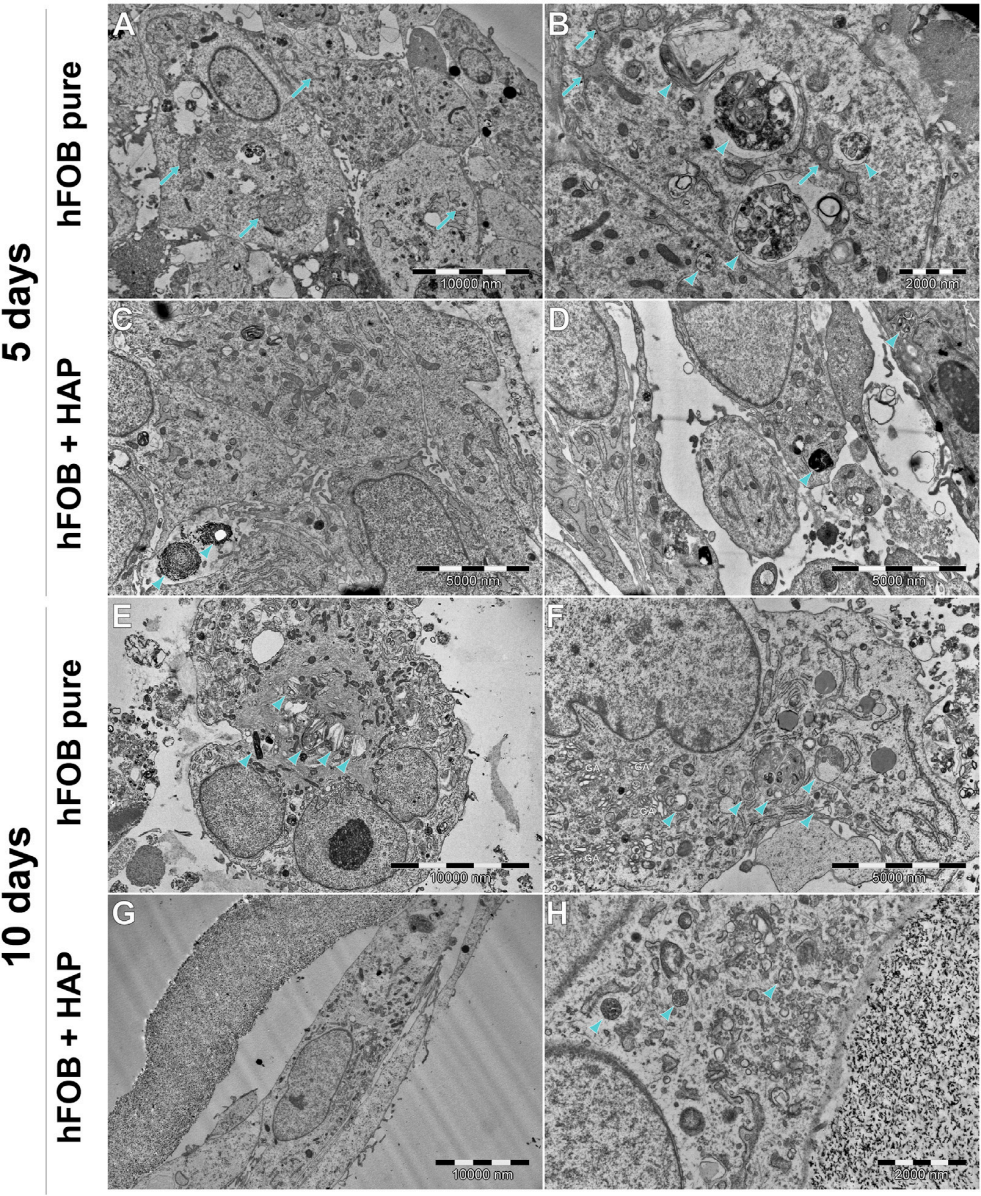
Ultrastructure of hFOB cells cultured on the scaffold, with or without HAp, after 5 or 10 days. **(A,B)** hFOB tend to form tight clusters, connected with numerous tight junctions. RER is extensive and wide, often forming a characteristic network (arrows). Multiple multilamellar bodies/early matrix vesicles can be found (arrowheads), indicating clearly on intensive mineralization. **(C,D)** hFOB with internalized HAp (arrowhead). **(E,F)** Most of the cells tend to have numerous multivesicular bodies and early/late matrix vesicles (arrowhead). Intense protein synthesis and secretion are indicated by a large amount of Golgi apparatus and extensive RER. **(G,H)** hFOB cultured with HAp for 10 days tend to be spindle-shaped, with less abundant multivesicular bodies, without clearly visible signs of mineralization. However, protein synthesis and secretion are still intense, as shown in **(H)**.

**FIGURE 12:**
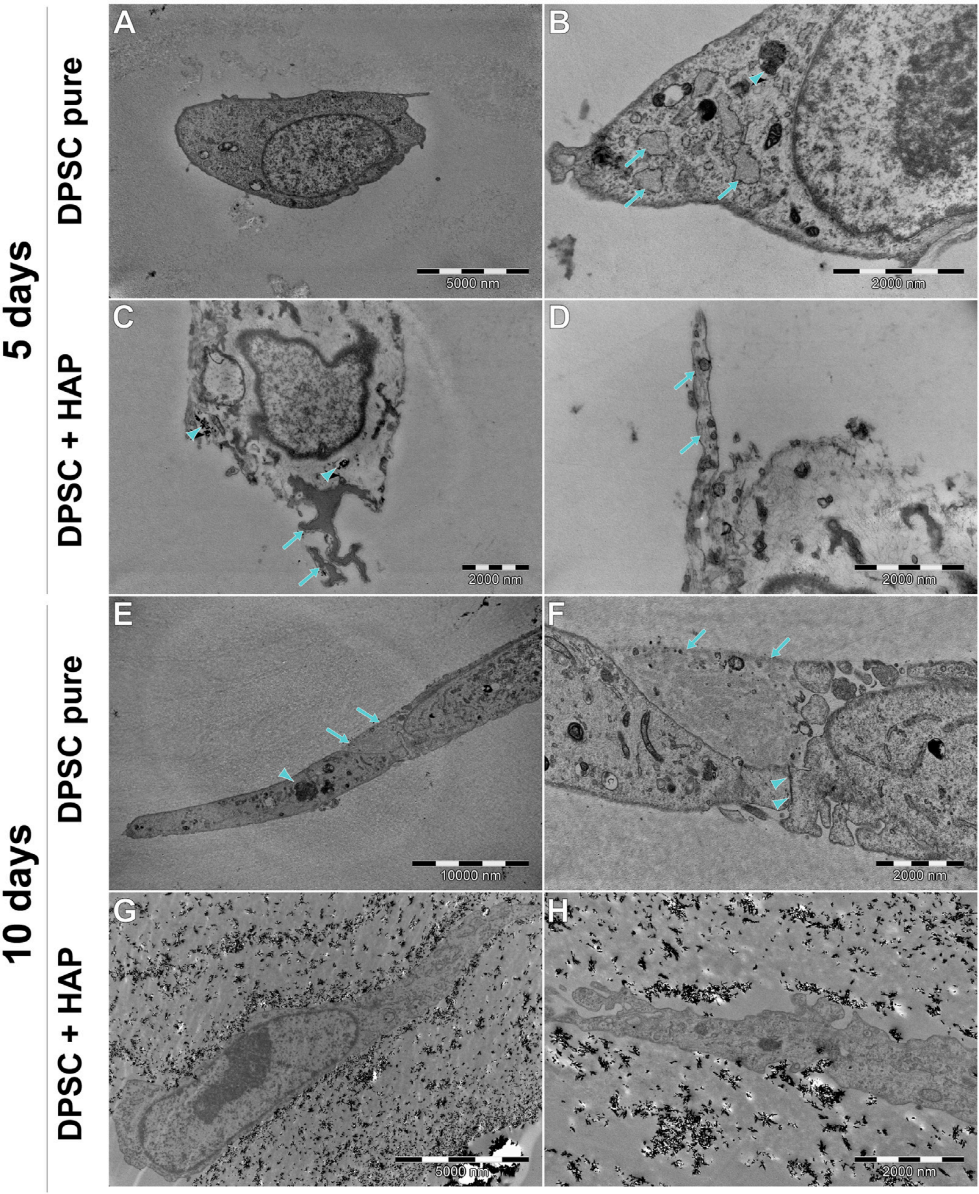
Ultrastructure of DPSCs cultured on the scaffold, with or without HAp, after 5 or 10 days. **(A,B)** Most of the DPSCs (PURE) are small, round cells, without extensive RER (arrows), although it is wider than that in DPSC cultured without the scaffold (see Figure 11). A small amount of electron-dense multivesicular bodies (arrowhead) can be found. **(C)** DPSCs cultured with HAp tend to internalize this mineral (arrowhead). Some of them tend to (putatively) initiate the mineralization processes (arrows). **(D)** Some DPSCs extend long protrusions with prominent fibers (arrows). **(E,F)** DPSCs surrounding the scaffold fragment are connected to each other by tight junctions (arrowheads). Cells also tend to be spindle-shaped with long protrusions. **(G,H)** Cells surrounded tightly by the scaffold modified with HAp. Cells tend to be spindle-shaped, with cytoplasm similar to the cells described previously.

Figure 10 shows a comparison of the hFOB 1.19 and DPSC lines cultured without a scaffold, Figure 11 shows the ultrastructure of the hFOB 1.19 cells cultured on a scaffold modified with or without HAp for 5 and 10 days, and Figure 12 shows the same but for DPSCs instead of hFOB 1.19 cells.

In general, the ultrastructure of the hFOB 1.19 cell lines cultured without scaffolds or HAp shows no signs of increased activity—in most of them, the visible rough endoplasmic reticulum (RER) is poorly developed, few organelles are visible, and they grow separately without visible junctions. When cultured on scaffolds (see Figure 11), their ultrastructure changes dramatically—numerous tight junctions between neighboring cells are present, forming large cell clusters. Numerous signs of increased secretory activity are visible, including well-developed Golgi apparatus, RER, and large amounts of multilamellar and multivesicular bodies. In some cells, matrix vesicles are present with visible crystals, presumably of hydroxyapatite which is typical for osteoblasts (Rohde and Mayer, 2007). Notably, the cells cultured for 10 days on scaffolds modified with HAp are visibly elongated, still secretionally active but with less numerous matrix vesicles.

DPSCs show a clearly different ultrastructure, resembling rather odontoblasts than osteoblasts (Sasaki and Garant, 1996; Rohde and Mayer, 2007). Vesicles produced by DPSCs show significantly different morphology (they are much smaller and electrons denser than those visible in hFOB 1.19 cells) typical of odontoblasts, and the organelles associated with secretory activity are less numerous. DPSCs found in scaffolds tend to fit into tight crevices and have an elongated shape. Furthermore, only single or two neighboring cells could be found, with no evidence of large cluster formation.

### 3.7 Droplet digital PCR

**FIGURE 13:**
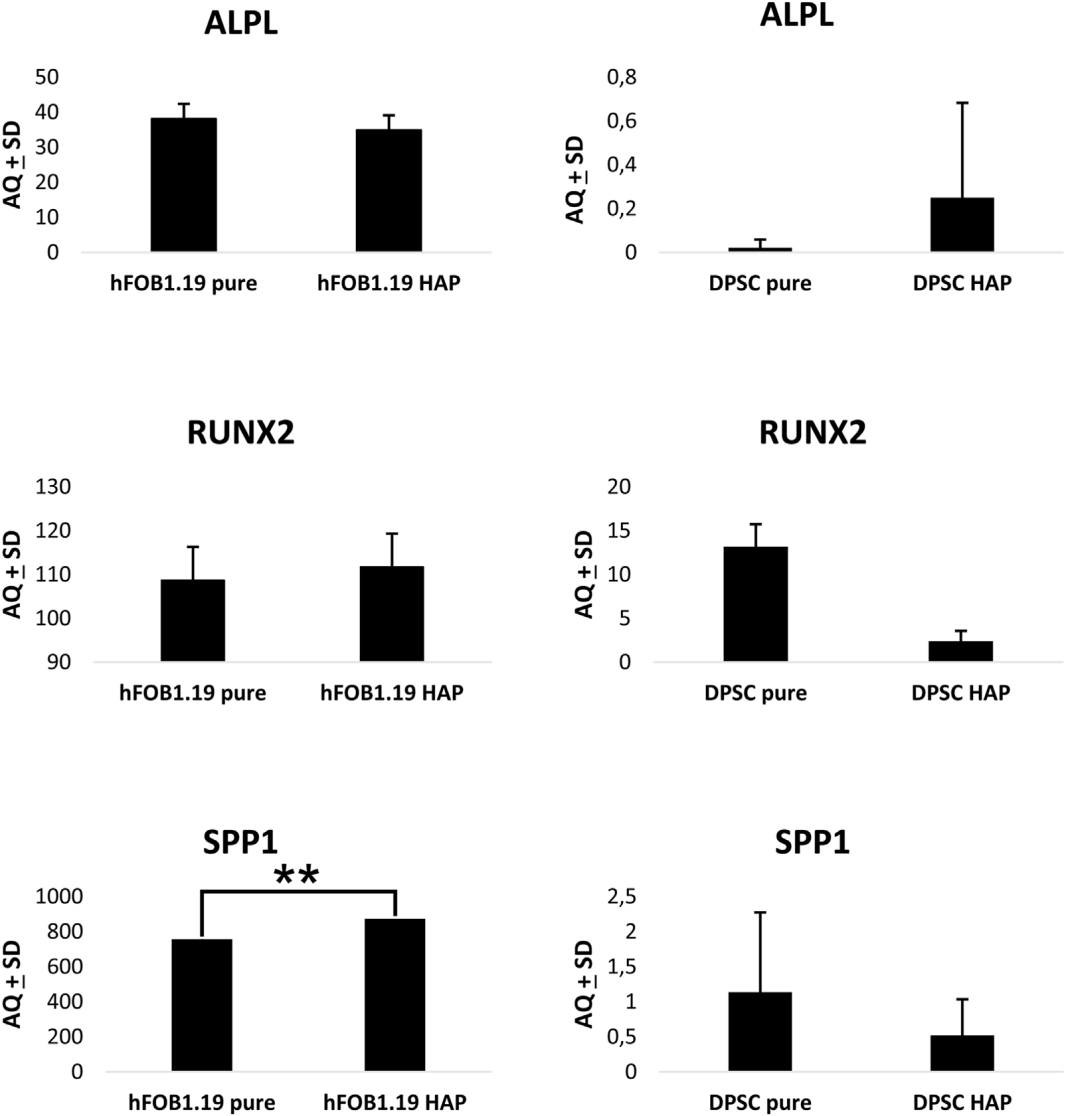
Droplet digital PCR results for osteogenic marker gene expression in DPSCs and hFOB 1.19 cells cultured on scaffolds: ALPL (alkaline phosphatase), RUNX2 (Runt-related transcription factor 2), and SPP1 (osteopontin). One-way ANOVA with Tukey’s *post hoc* tests for multiple comparisons was performed.

Osteogenic marker gene expression was assessed by performing ddPCR with the use of specific primers and probes for ALP (alkaline phosphatase), RUNX2 (Runt-related transcription factor 2), and SPP1 (osteopontin), as shown in Figure 13. Analytical studies were conducted after 10 days of cell culture on a scaffold. The expression of ALP mRNA follows the activity of ALP, and the expression of ALP, as a reliable marker of early osteogenesis/odontogenesis, was confirmed by the staining method. The results showed that the DPSC expression of ALP mRNA was higher on the HAp-modified chitin scaffold than on the pure chitin scaffold. In the case of hFOB 1.19 cells, the study showed a higher level of ALP mRNA expression on the pure scaffold without modification, and ALP expression was significantly higher compared to that in DPSCs. Similarly, in the case of RUNX2 expression in hFOB 1.19 cells, the highest level was observed on the HAp-modified chitin scaffold, while a significantly lower level was demonstrated in DPSCs on the same scaffold. RUNX2 is a marker of the late, final stage of differentiation of mesenchymal stem cells into osteoblasts or odontoblasts.

The reduction in RUNX2 transcriptional activity may be related to the maintenance of DPSCs in an undifferentiated state and indicates that the differentiation of DPSCs was not complete. However, by expanding the number of cells, it is possible to increase their osteogenic potential, as in BM-MCS. The level of SPP1 (osteopontin) mRNA expression in DPSCs was significantly higher on the pure chitin scaffold compared to the HAp-modified chitin scaffold in contrast to hFOB 1.19 cells where an increase in the expression of SPP1 mRNA activity was observed on the HAp-modified scaffold. The result obtained in ddPCR showed the biological activity of chitin scaffolds and stimulation of DPSCs in the direction of osteoblast- or odontoblast-like cells by chitin scaffolds modified with HAp. Furthermore, hFOB 1.19 cells also indicate the stimulation of osteoblast cell activity, due to RUNX2 and osteopontin expression. The results obtained with the ddPCR method for DPSCs on chitin scaffolds are consistent with the expression of ALP observed in these cells; increased expression of early-stage differentiation markers at the mRNA and protein levels indicates that the chitin scaffold modified with HAp stimulates DPSCs at an early stage of differentiation into osteoblasts or odontoblasts. Furthermore, the research conducted showed that differentiated osteoblasts (hFOB 1.19 cells) express typical and physiological markers.

## 4 Discussion

In nature, chitin is a widely distributed structural polysaccharide (second most abundant after cellulose). Its exceptional properties, such as well-developed chemical structure, biocompatibility, biodegradability, and renewability, make it an interesting candidate for several modern applications (Schubert et al., 2019). However, the limitations resulting from the structural properties of chitin, such as its significantly difficult solubility, limit its commercial application. The fundamental work of Barzic and Albu (2021); Ehrlich et al., 2007 shows in detail the cohesive interaction of crustacean chitin with blood components, such as erythrocytes, fibrinogen, blood fluid, albumin (beneficial for preventing side effects), and microbes (like *Streptococcus oralis*, *Escherichia coli*, *Shigella dysenteriae*, *Staphylococcus epidermidis*, and *Actinomyces naeslundii*), which proves their bacteriostatic properties. These results confirm the potential biomedical utility of chitin.

As observed, a positive Ws (adhesion) value for collagen can definitely influence a successful cell culture. However, in addition to the biocompatibility of the material, other parameters must be met to produce an effective scaffold. Commercial chitin is isolated from fungal biomass or crab, lobster, shrimp, and crayfish shells and can be obtained in the form of granules, sheets, or powder. However, these forms need to be further developed to create an effective scaffold. Three-dimensional α-chitin skeletal scaffolds derived from marine demosponges have been recognized as excellent supports for cell attachment and proliferation in their native form. However, only a few cell types have been developed for seeding: chondrocytes (Steck et al., 2010), human mesenchymal stem cells (Machałowski et al., 2021a), cardiomyocytes (Huang, 2012; Mazzoni et al., 2021), fibroblasts, keratinocytes, neurons (Machałowski et al., 2021a), and osteoblasts. All these publications show biocompatibility and non-cytotoxic properties of chitin scaffolds. This is also confirmed by the research we have conducted on DPSCs and hFOB 1.19 osteoblasts (Machałowski et al., 2021b).

In this study, for the first time, a poriferan chitinous scaffold isolated from *A. fistularis* was developed as a support for the cultivation of DPSCs in comparison to hFOB 1.19 cells. In our investigation, the pure chitinous scaffold created a favorable environment for the adhesion of DPSCs and hFOB 1.19 cells and supported cell proliferation and differentiation. The studies conducted showed a large number of cells with the typical morphology on the scaffold surface. These results confirmed the non-cytotoxic character and good biocompatibility of chitin scaffolds.

Previous studies have shown the mechanical properties of α-chitin scaffolds (Machałowski et al., 2022; Duminis et al., 2023). Machałowski et al. (2021b) demonstrated that the Young’s modulus of chitin nanofibers ranged from 0.1 kPa to 1 MPa. The ideal bone/dental engineering scaffold should provide sufficient stiffness to mimic the native ECM. In research by Huang (2012), human mesenchymal stem cells (hMSCs) exhibit osteogenic differentiation when cultured on a scaffold with a stiffness similar to the nascent bone >34 kPa. Another important factor in tissue formation is the porous structure of biomaterials. It affects migration, proliferation, nutrient diffusion, mass transport, and vascularization. The minimum requirement for the pore size of biomaterials used for bone regeneration should be approximately 100 μm (Mazzoni et al., 2021). Previous studies by Machałowski et al. (2021b) have shown that the average pore diameter of the poriferan chitinous scaffold of *A. fistularis* is 298 µm and the fiber thickness is approximately 98 µm.

To make the scaffold even more similar to the native bone tissue, the author decided to use hydroxyapatite and created a biocomposite with α-chitin skeletal scaffolds due to its excellent osteoinductive and osteoconductive properties. Due to its good integration with hard and soft tissues, HAp is widely used in tissue engineering and regenerative medicine, and its porosity (Li et al., 2020) has been shown to result in rapid blood vessel revitalization and graft revascularization. Our latest experiences showed that the addition of HAp improved mechanical properties of chitin–HAp composites (Machałowski et al., 2021a). Unmodified HAp exhibits low mechanical strength, so it is recommended to combine it with another material to ensure adequate stiffness of the structure (Michel et al., 2015), which can modulate the osteogenic potential and mechanical properties of the medium.

Previous studies have shown that scaffolds synthesized by the coprecipitation reaction of chitosan with hydroxyapatite stimulate BMSCs to form mineralized nodules on the scaffold. It was also shown that scaffolds with higher concentrations of HAp induced greater mineralization and formation of a structure similar to the native bone tissue (Zak et al., 2023). Our results confirmed that synthesized HAp and chitin are suitable for cell adhesion. The 3D chitinonus scaffold from *A. fistularis* modified with HAp provided an environment that supported both DPSCs and hFOB 1.19 cells. Various staining techniques, including crystal violet and neutral red, demonstrated that the appropriate physicochemical parameters and the porosity of the scaffold provided structural support in cell culture and ensured the interaction, adhesion, and proliferation of cells on the surface of the material while maintaining the typical cell morphology. No cytotoxic properties were found to affect the biological safety of the chitin–HAp and collagen composite scaffolds (Xing et al., 2021). As observed, α-chitin skeletal scaffolds modified with HAp have a stimulatory effect on the cells. DPSCs on modified scaffolds were able to initially differentiate into osteoblast- or odontoblast-like cells. Expression of ALP, RUNX2, osteopontin, and Alizarin S are typical markers of mesenchymal stem cell differentiation into osteoblasts or odontoblasts. The studies performed showed the appearance of an early step in the differentiation of DPSCs into osteoblasts or odontoblasts, and ALP expression is a marker of this process. However, the results obtained by TEM methods suggest rather that differentiation into odontoblasts is more likely. These results clearly demonstrate the biological potential of chitin scaffolds in the field of cell differentiation. DPSCs have the potential to differentiate into odontoblasts or osteoblasts, as shown in the work of Son et al. (2021) and Wang et al. (2013). It is worth noting that osteoblasts and odontoblasts are closely related cells, and similarities exist also between dentin and bone (Son et al., 2021), so the chitin scaffold used to regenerate both may be congenial. In our research, we observed DPSCs and osteoblast cells at a similar time after 5 and 10 days of cell culture on chitin scaffolds. This, not a prolonged culture of cells on scaffolds, especially dental stem cells, provides us more information about the initial steps of cells and scaffold interactions. These direct interactions were analyzed in this step of research.

Despite research conducted on animal models, there are difficulties in the therapeutic application of the tissue engineering triad to reconstruct the pulp–dentin complex (Mao et al., 2012). Attempts have been made to regenerate the pulp–dentin complex with various combinations of scaffolds and DPSCs, including collagen-derived materials (Iohara et al., 2016; Iohara et al., 2020), hyaluronic acid gel (Zhu et al., 2018), nanofibrous spongy microsphere poly (L-lactic acid) (Kuang et al., 2016), platelet-rich plasma (Zhu et al., 2014), and chitosan hydrogel (Palma et al., 2017; El Ashiry et al., 2018). The strategy of injectable biomaterials in the regeneration of the pulp–dentin complex is preferable due to their ability to adapt to the complex anatomy of root canal systems (Huang et al., 2009). The chitosan hydrogel was investigated as a natural scaffold for the regeneration of pulp–dentin-like tissues in permanent teeth with apical periodontitis by El Ashiry et al. (2018); Kuang et al. (2016). They used scaffolds seeded with DPSCs in the canine model. It was shown that pulp-like tissue and dentin-like tissue along the root canal walls could regenerate after 4 months. Palma et al. (2017) conducted experiments with chitosan scaffolds in endodontic treatment and showed that the regeneration of a pulp–dentin complex was not improved after the addition of chitosan scaffolds during canine endodontic regeneration procedures. Research on multifunctional nanocomposite scaffolds was also conducted by Hoctor et al. (2013). They showed that the combination of chitosan and hydroxyapatite may be suitable for use as a bone substitute material as the samples contained a higher ratio of HAp, cross-linking agent, and photoinitiator solution. In our study, we showed that hFOB 1.19 cells form tight clusters on scaffolds, and the TEM study indicated the presence of numerous tight junctions and intensive mineralization. Its physical properties and biological activity stimulate selected cells to produce the biochemical components of bone.

Due to the similar origin of osteoblasts and odontoblasts, further detailed studies are required to evaluate the major markers of odontoblasts, including dentin sialophosphoprotein (DSPP) and dentin matrix protein-1 (DMP-1), to distinguish between osteogenic and odontogenic differentiation of DPSCs.

The results suggest that the chitinous scaffold modified with hydroxyapatite may be useful for bone tissue engineering and in dentin–pulp complex regeneration. However, further studies are needed to optimize its clinical potential.

## 5 Conclusion

Materials for tissue engineering applications, such as bone regeneration, have been studied for many years. Three-dimensional scaffolds should be biocompatible and not interfere with cell adhesion and differentiation. Many scientific reports indicate the beneficial effect of materials of natural origin on the directed growth and differentiation of stem cells. In this study, we reported that chitin scaffolds, especially those modified with HAp, have potential for DPSC adhesion and stimulation into osteoblasts or odontoblasts. It is worth emphasizing that the potential application site could stimulate DPSCs to completely differentiate, and the differentiated cells could proliferate and show biological activity. Our results indicate that chitin scaffolds, especially those modified with HAp, have the potential to be used in the field of regenerative dentistry. However, the fundamental question of the deeper understanding of the relationship between mechanical properties and DPSC sensitivity still remains unanswered and will require new approaches in future research.

## Data Availability

The original contributions presented in the study are included in the article/Supplementary Material; further inquiries can be directed to the corresponding author.
