# Supplementary figures and images for: Chitin scaffolds derived from the marine demosponge Aplysina fistularis stimulate the differentiation of dental pulp stem cells
Source: Front Bioeng Biotechnol. 2023 Nov 14;11:1254506. doi: 10.3389/fbioe.2023.1254506 (PMC10682193; doi:10.3389/fbioe.2023.1254506)

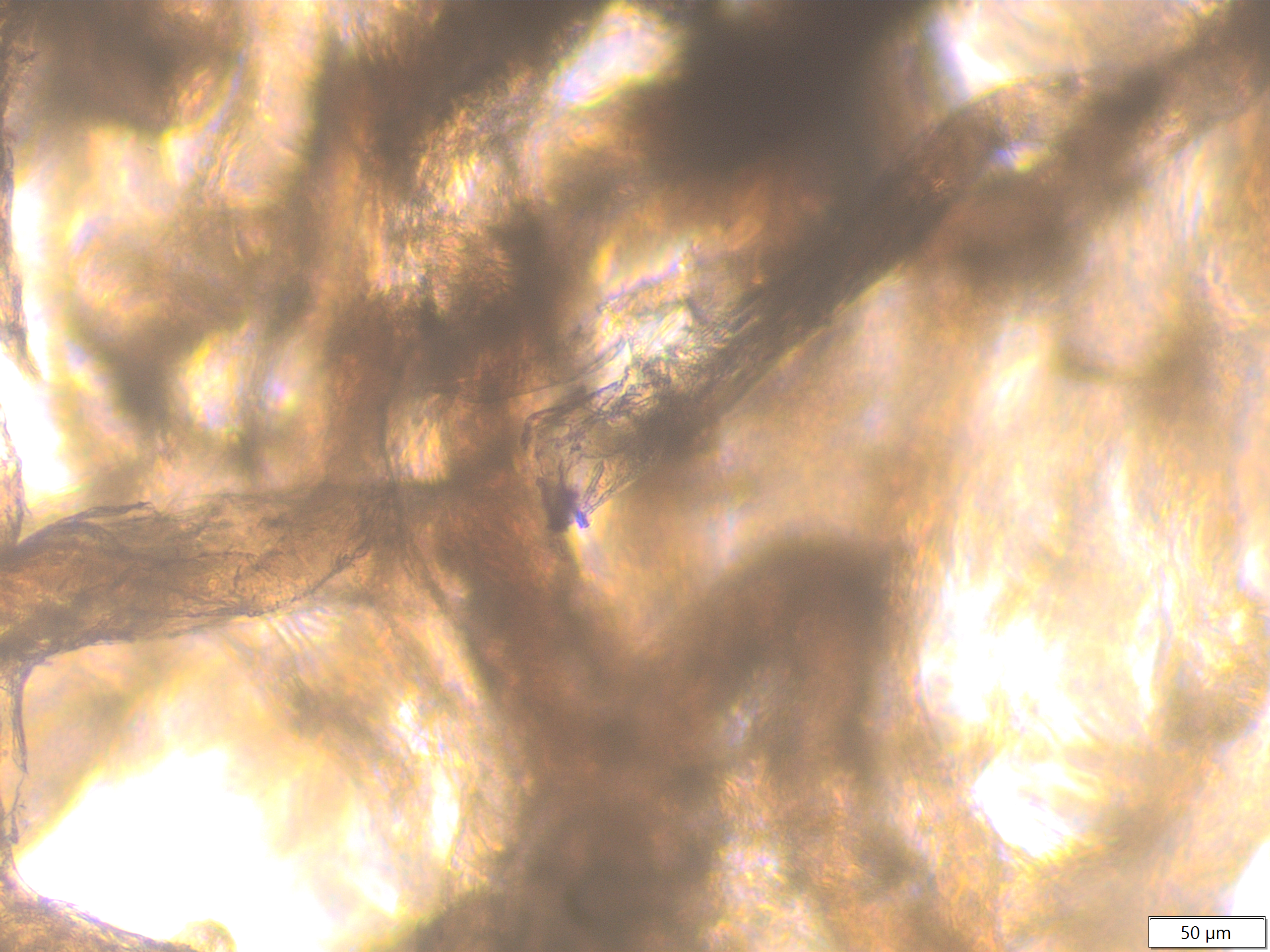

Supplement: Supplementary file 1 [file Image6.TIF]

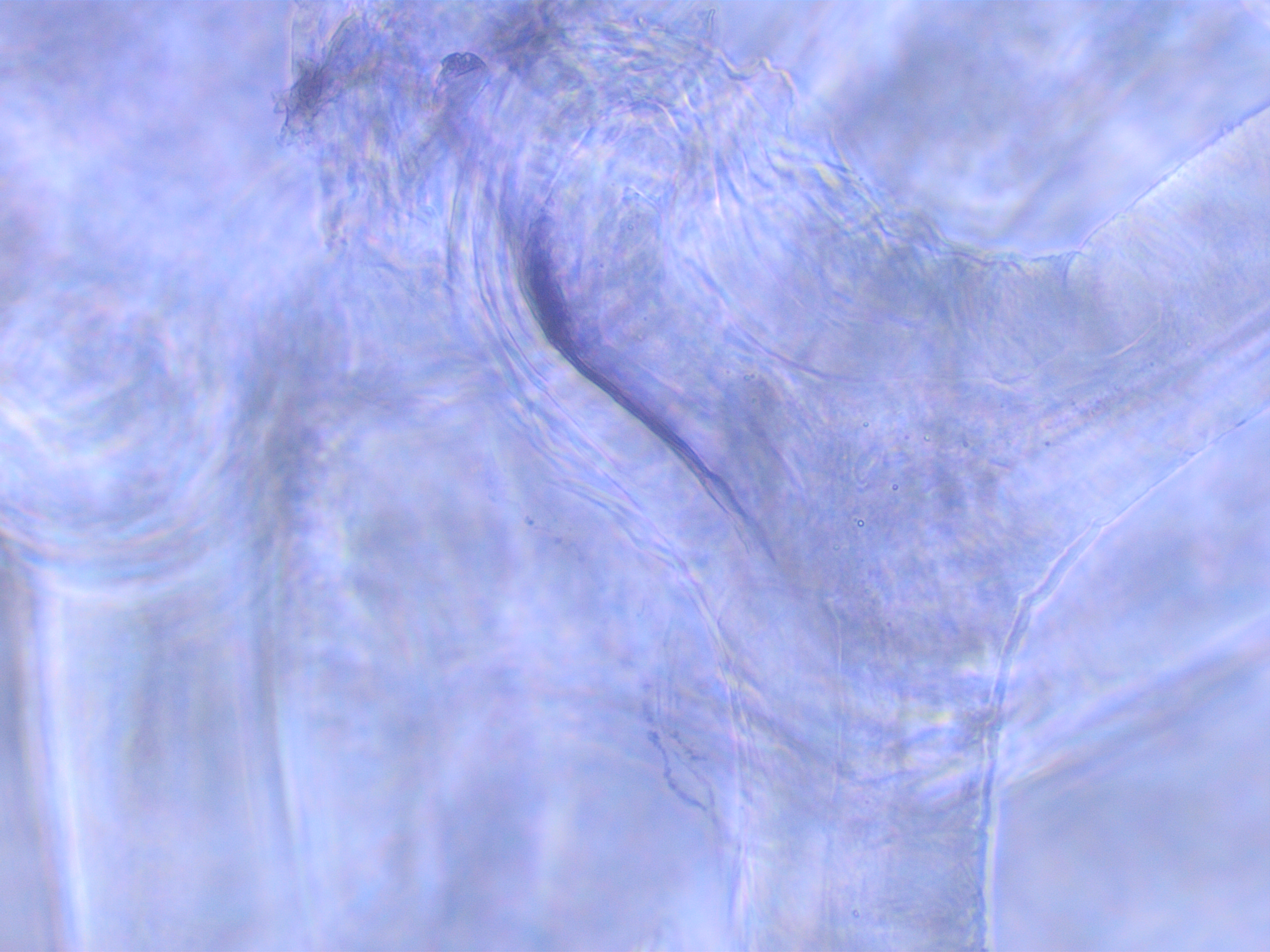

Supplement: Supplementary file 2 [file Image3.TIF]

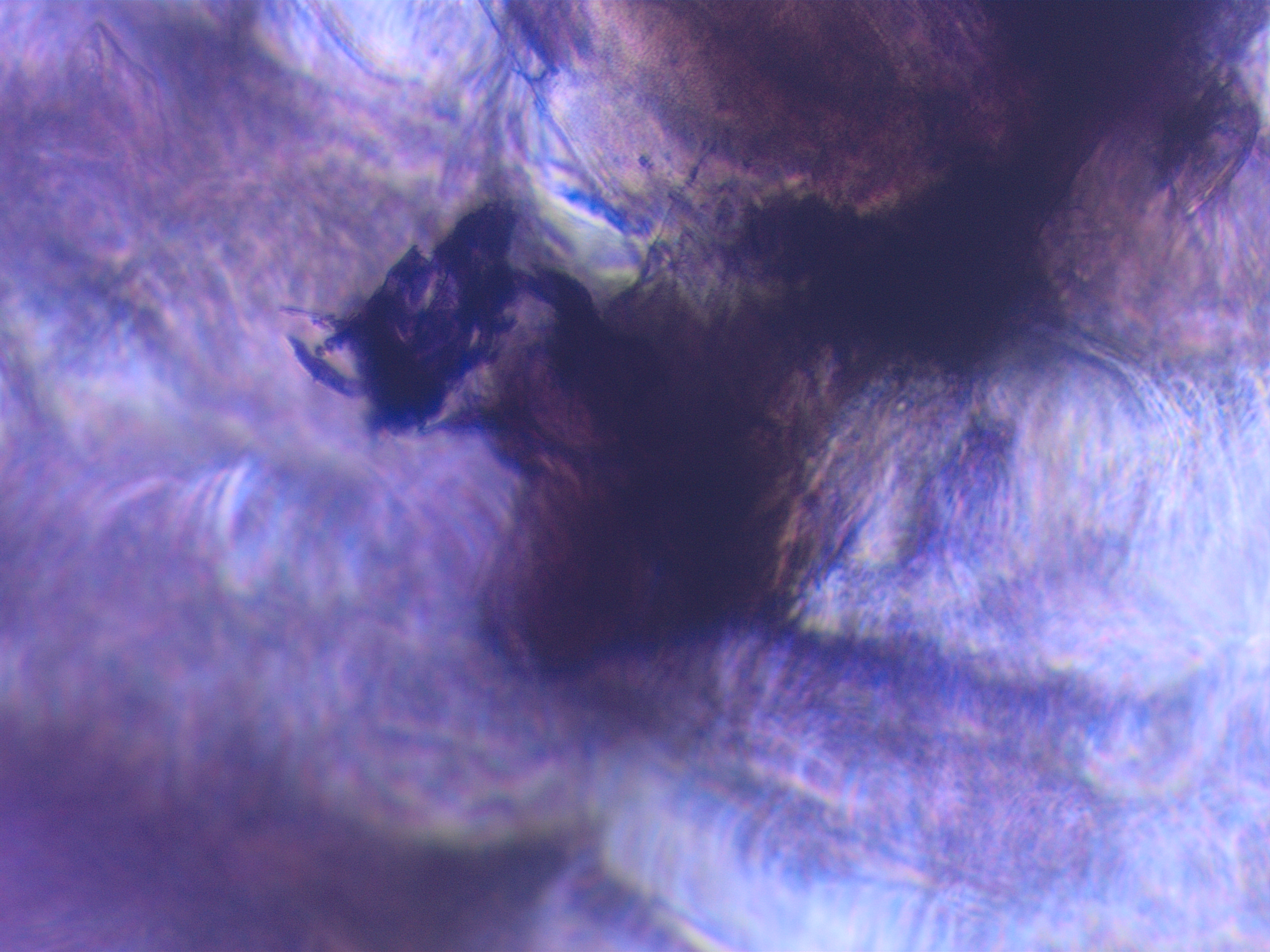

Supplement: Supplementary file 3 [file Image4.TIF]

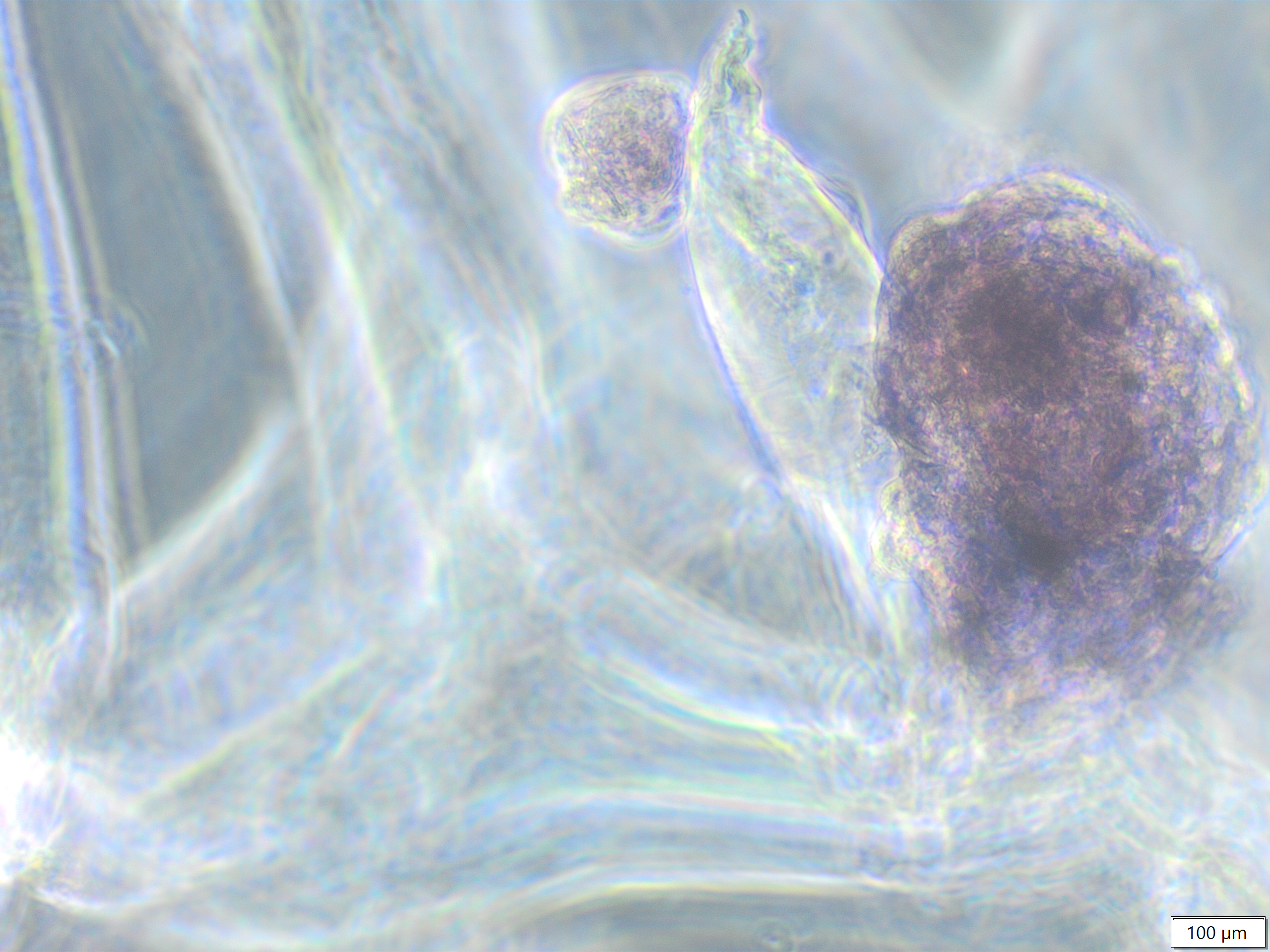

Supplement: Supplementary file 4 [file Image9.TIF]

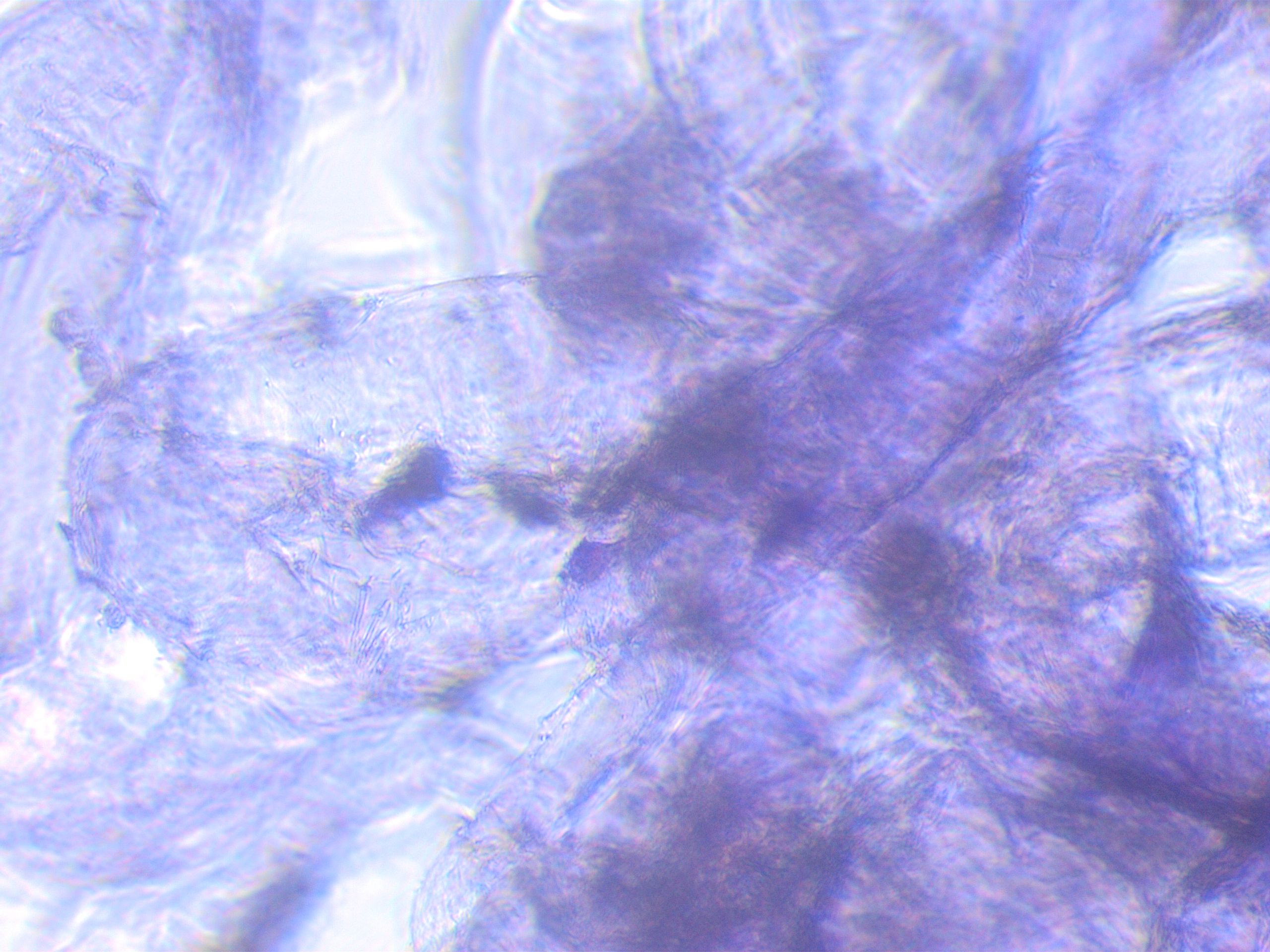

Supplement: Supplementary file 5 [file Image2.TIF]

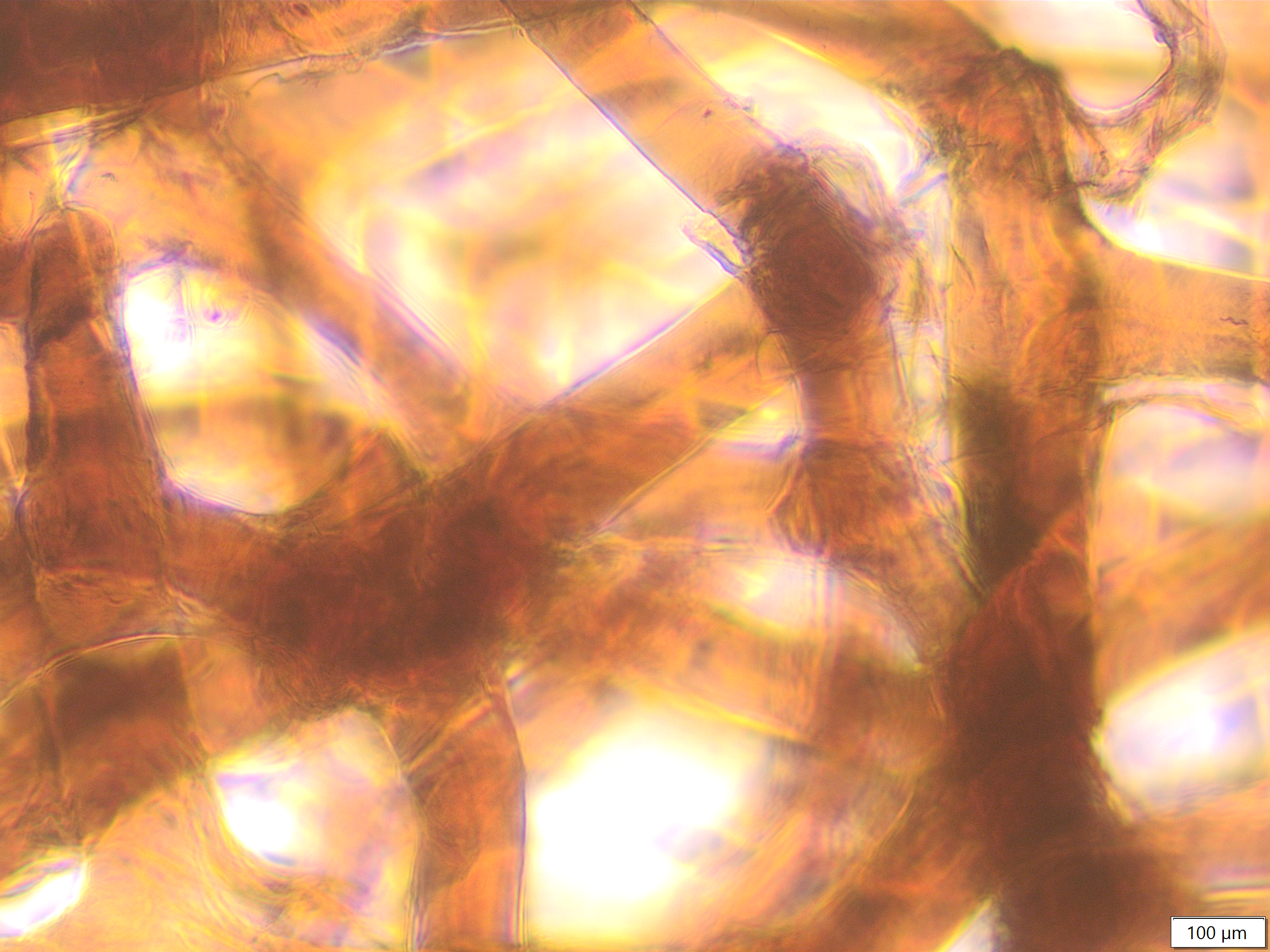

Supplement: Supplementary file 6 [file Image11.TIF]

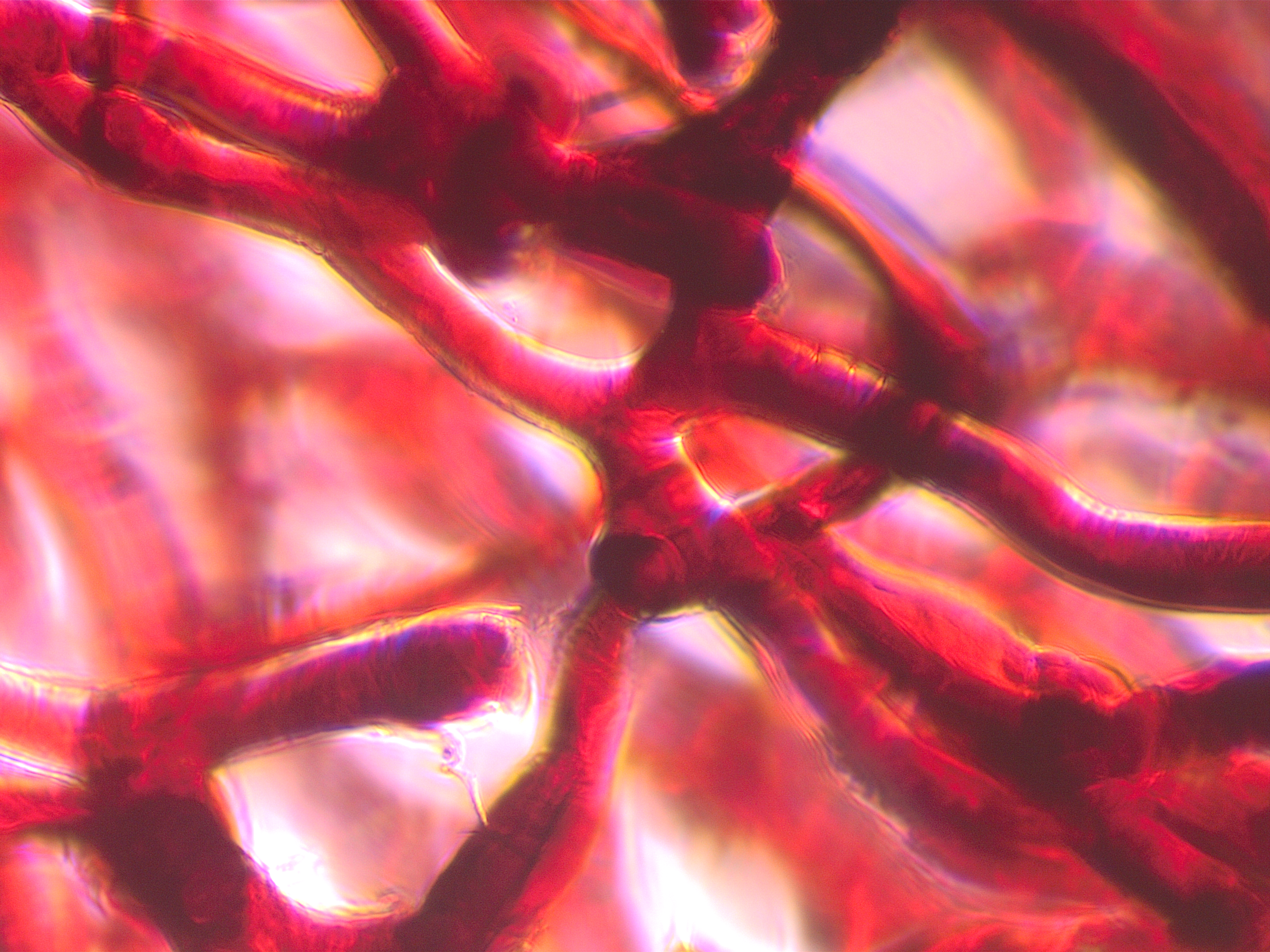

Supplement: Supplementary file 7 [file Image1.TIF]

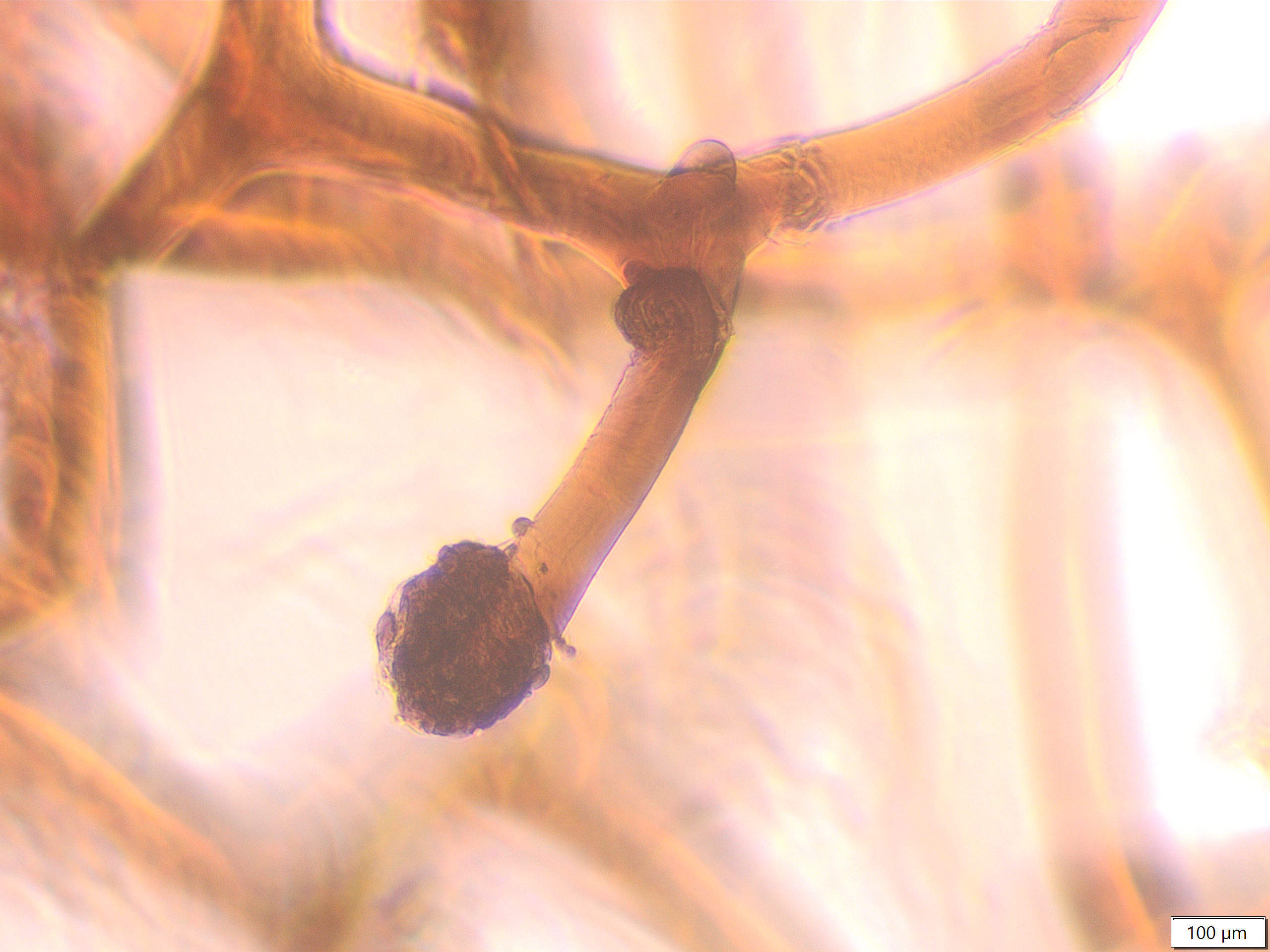

Supplement: Supplementary file 8 [file Image10.TIF]

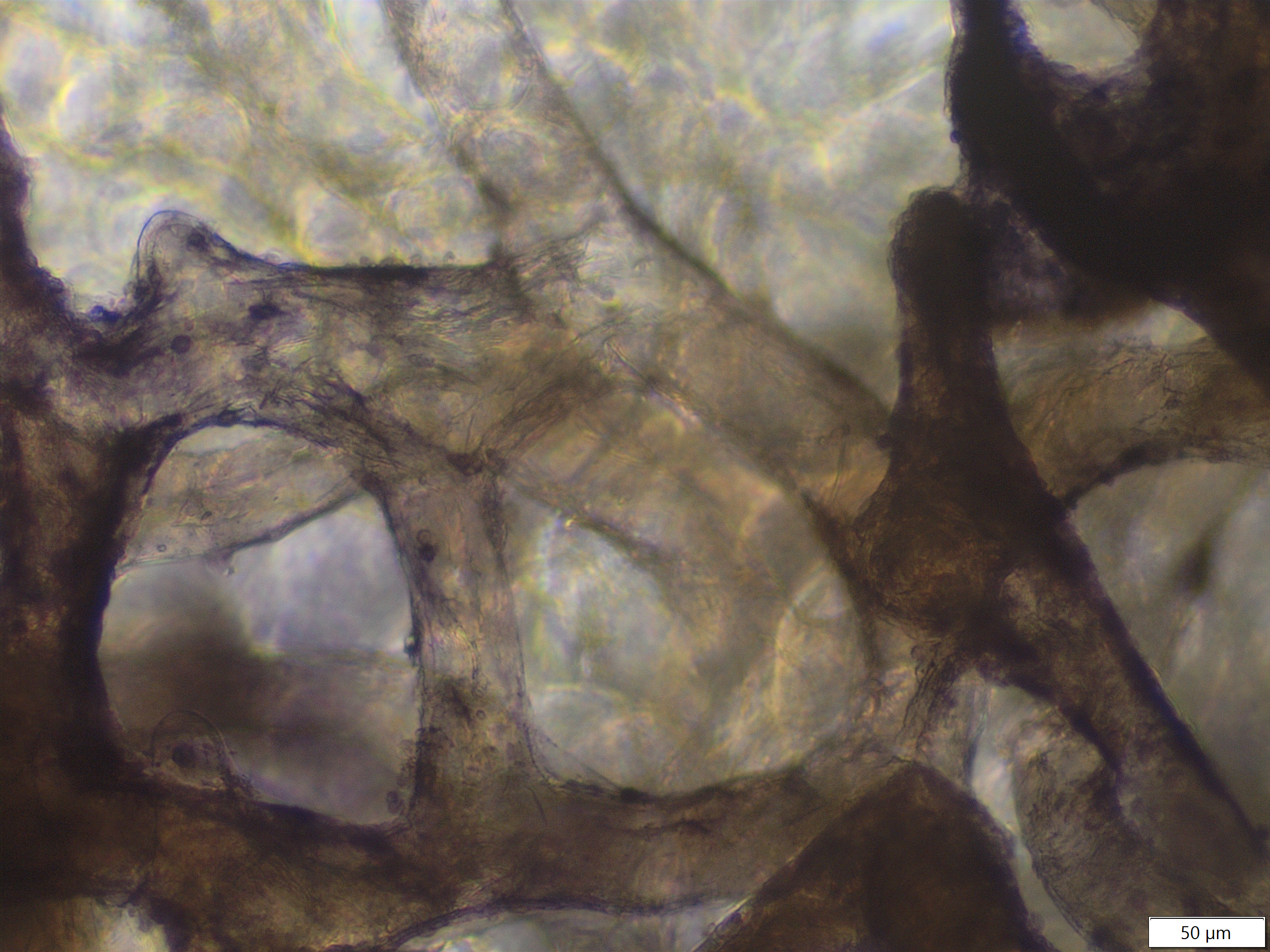

Supplement: Supplementary file 9 [file Image7.TIF]

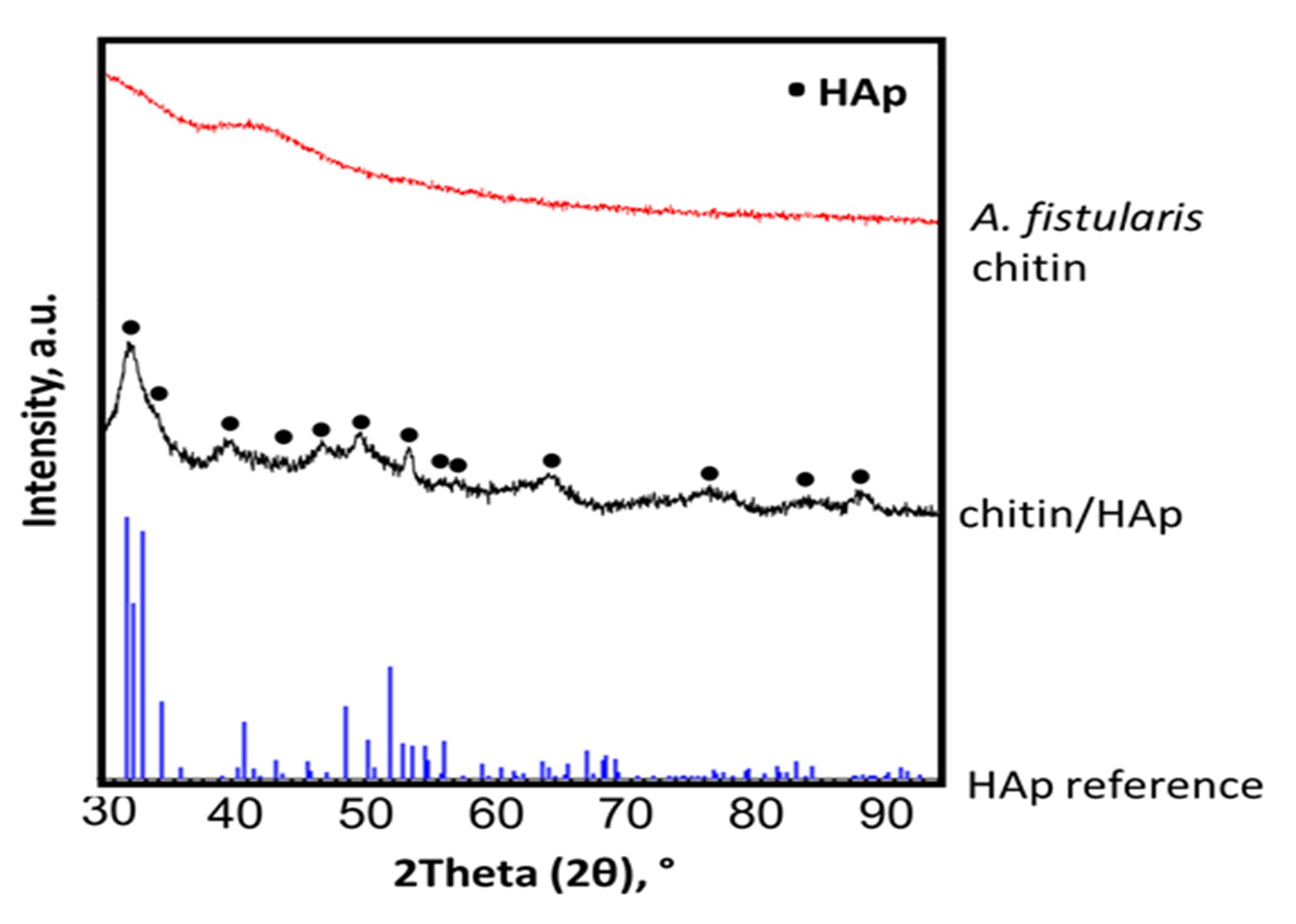

Supplement: Supplementary file 11 [file Image13.JPEG]

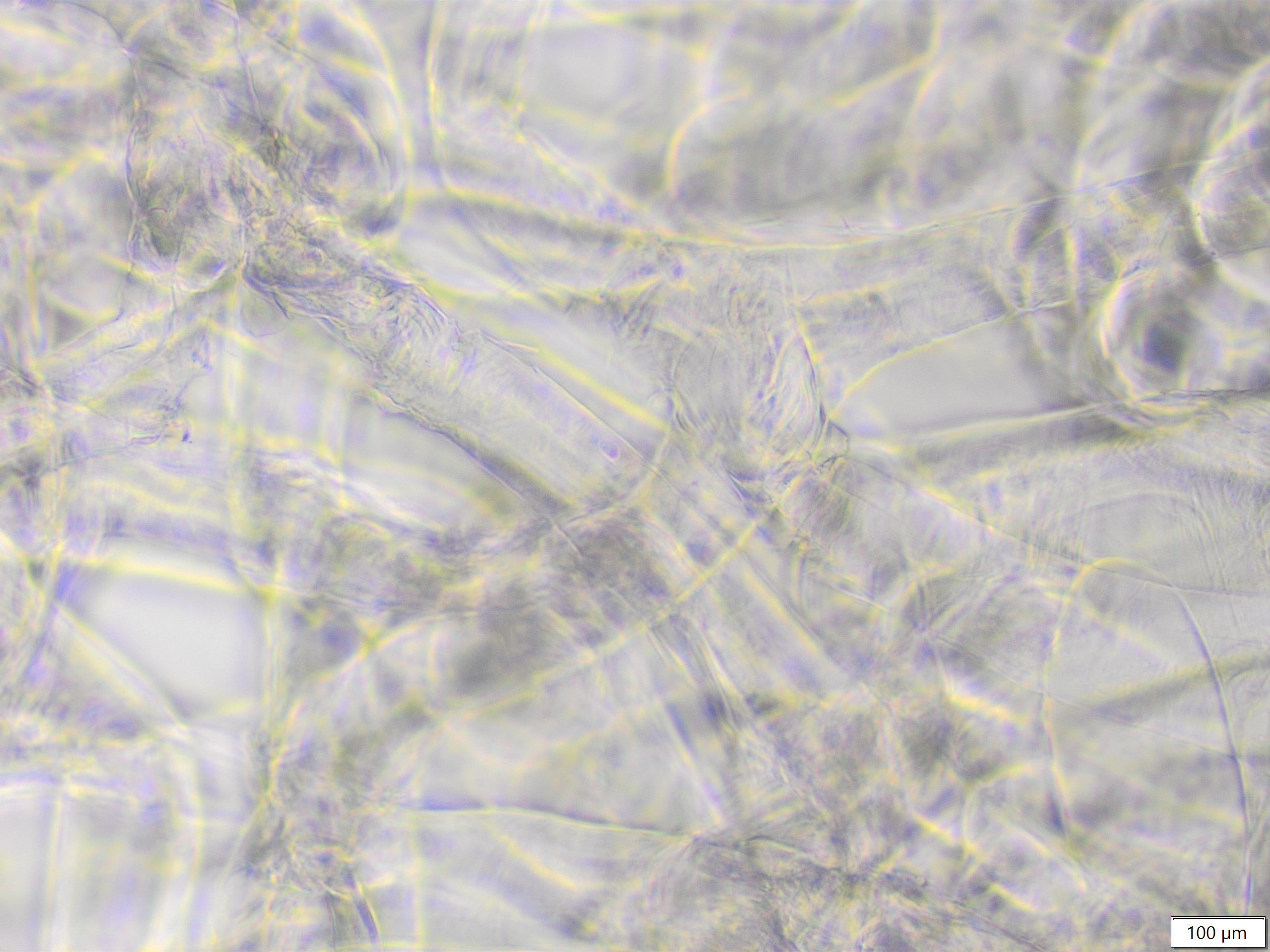

Supplement: Supplementary file 12 [file Image8.TIF]

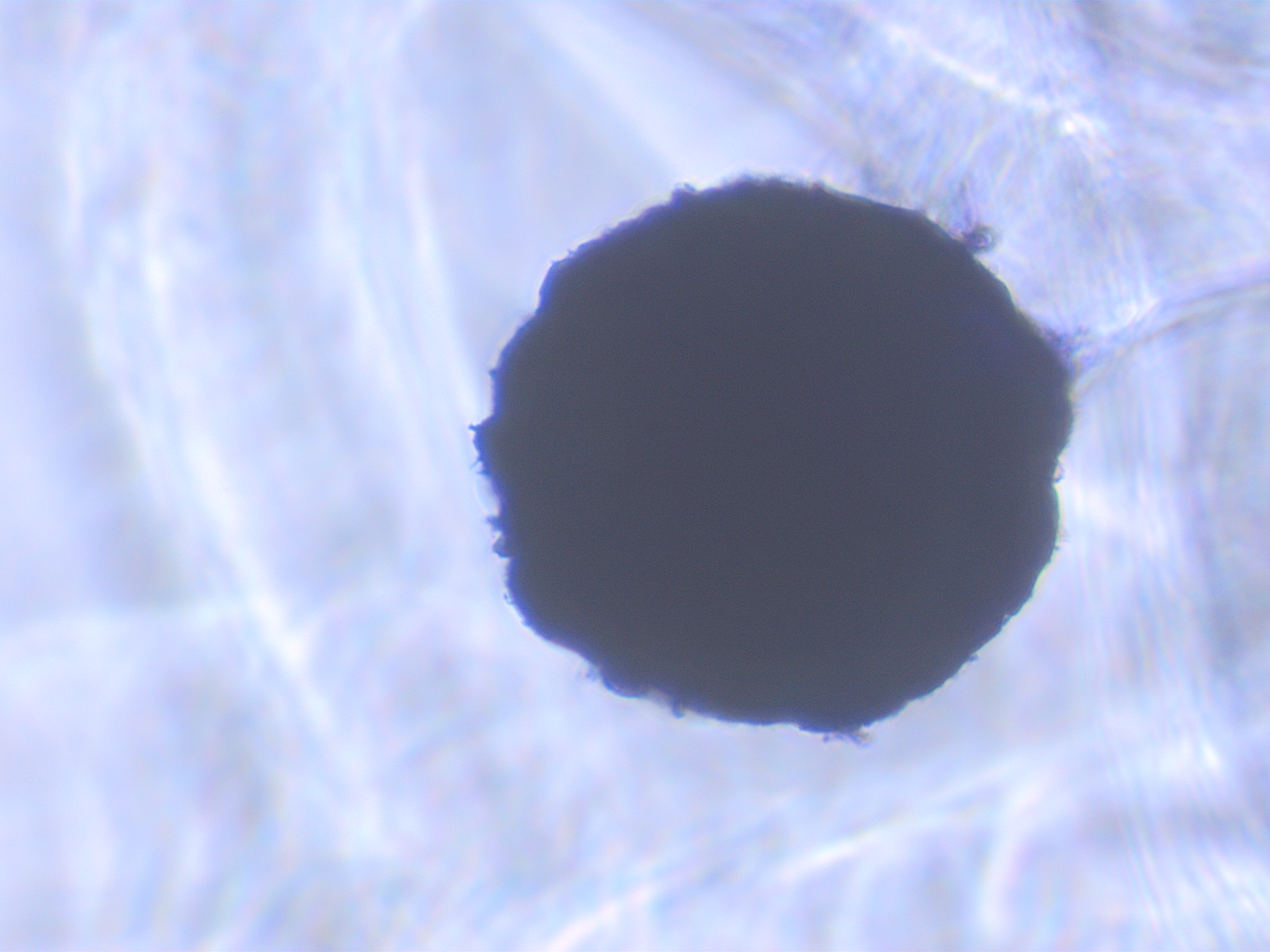

Supplement: Supplementary file 13 [file Image5.TIF]

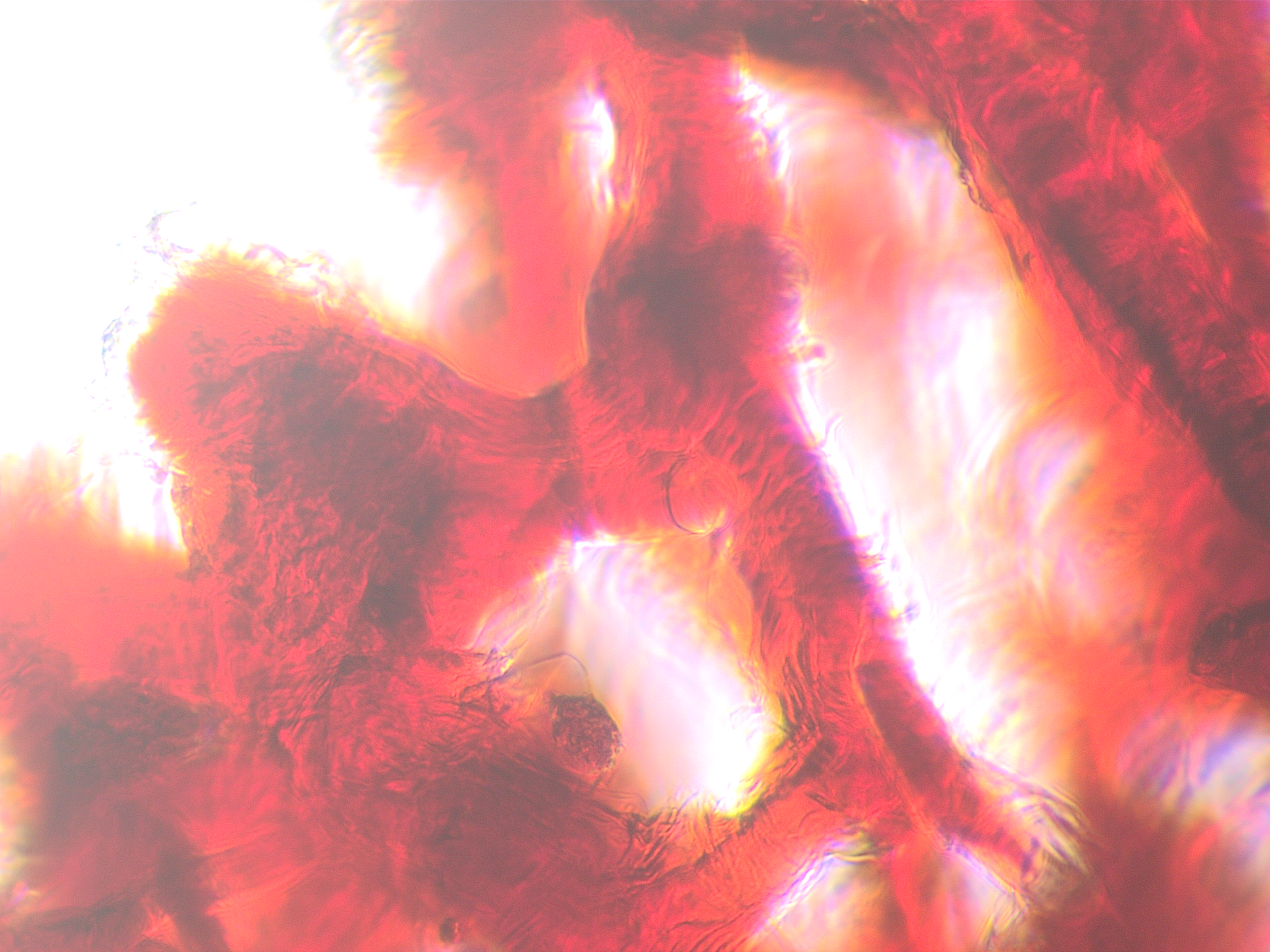

Supplement: Supplementary file 14 [file Image12.TIF]
